# Femtosecond‐Laser Synthesized PtBi Nanoalloys for Efficient Methanol Oxidation in Hybrid Electrolysis

**DOI:** 10.1002/advs.202510123

**Published:** 2025-08-18

**Authors:** Xianze Zhang, Zikang Su, Chen Zhang, Zhiyi Sun, Lan Jiang, Zhi Wang, Ruichen Lu, Qimiao Zhu, Shucheng Shi, Yunhong Luo, Yang Gu, Zhi Liu, Wenxing Chen, Hui Zhang, Xueqiang Zhang

**Affiliations:** ^1^ Laser Micro/Nano‐Fabrication Laboratory School of Mechanical Engineering Beijing Institute of Technology Beijing 100081 China; ^2^ Energy & Catalysis Center School of Materials Science and Engineering Beijing Institute of Technology Beijing 100081 China; ^3^ School of Physical Science and Technology ShanghaiTech University Shanghai 201210 China; ^4^ Center for Transformative Science ShanghaiTech University Shanghai 201210 China; ^5^ Shanghai Synchrotron Radiation Facility Shanghai Advanced Research Institute Chinese Academy of Sciences Shanghai 201204 China; ^6^ National Key Laboratory of Materials for Integrated Circuits Shanghai Institute of Microsystem and Information Technology Chinese Academy of Sciences Shanghai 200050 China

**Keywords:** femtosecond (fs) laser, *in‐situ* spectroscopy, methanol oxidation reaction (MOR), platinum bismuth (PtBi) nanoalloy, seawater electrolysis

## Abstract

Hydrogen production through electrochemical seawater splitting is challenged by the energy‐intensive oxygen evolution reaction and the competing chlorine evolution reaction. To overcome these obstacles, ligand‐free platinum bismuth (PtBi) alloy nanoparticles (≈2 nm) are synthesized via femtosecond laser liquid ablation under nonequilibrium conditions, yielding metastable structures with tunable elemental compositions populated with defects. The Pt_4_Bi/C catalyst excels in alkaline methanol oxidation reaction (MOR), delivering a mass activity of 17.7 A mg^−1^_pt_ (11.5 times higher than 20% Pt/C) and a specific activity of 54.9 mA cm^−2^. *In‐situ* Fourier transform infrared spectroscopy and ambient pressure X‐ray photoelectron spectroscopy reveal a CO‐free pathway enabled by Bi, reducing catalyst poisoning. Density functional theory calculations show that PtBi─O lowers d‐band center of Pt, weakening the adsorption of *CO, promoting the adsorption of *OH, and lowering the energy barrier from *CHO to *HCOOH. As an example, a hybrid MOR–hydrogen evolution reaction (HER) electrolyzer demonstrates reduced voltage, suppresses side reactions, improves catalyst durability, achieves 545 mV at 10 mA cm^−2^, and maintains stability for 54 h below 1.1 V in natural seawater. This study demonstrates the efficacy of PtBi nanoalloys in efficient MOR catalysis for hybrid electrolysis systems toward sustainable hydrogen production.

## Graphical abstract

Platinum bismuth (PtBi) catalysts, synthesized ligand‐free via femtosecond laser ablation, excel in methanol oxidation reaction with 11.5 times higher mass activity than Pt/C. Bi enhances Pt's efficiency and durability, enabling stable seawater electrolysis for hydrogen production. This breakthrough advances sustainable energy solutions.

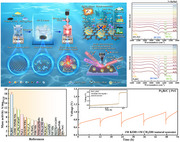

## 1 Introduction

Hydrogen (H_2_) is a promising energy carrier for carbon neutrality due to its high energy density (142 MJ kg^−1^) and zero carbon emission while used as a fuel.^[^ ^1^ ^]^ Electrochemical water splitting offers a sustainable route for large‐scale H_2_ production, but it is hindered by the energy‐intensive oxygen evolution reaction (OER) at the anode, which requires high overpotentials due to sluggish kinetics.^[^ ^2^, ^3^ ^]^ Seawater, as an abundant resource, presents a cost‐effective alternative for electrolysis, though it introduces additional challenges such as the competing chlorine evolution reaction (CER) and the corrosion of working electrodes under basic and high concentration ions.^[^ ^4^ ^]^ To mitigate these issues, replacing OER with the electrooxidation of small molecules, such as methanol, can lower the operating voltage and enhance system efficiency.^[^ ^5^, ^6^, ^7^, ^8^, ^9^, ^10^ ^]^ Methanol has a significantly lower oxidation potential (0.016 V vs RHE), reducing the operating voltage and mitigating the risks of H_2_/O_2_ explosion and CER interference.^[^ ^11^, ^12^ ^]^

Methanol oxidation reaction (MOR) is particularly advantageous for hybrid water electrolysis due to its low oxidation potential and ability to produce pure H_2_ under ambient conditions without the generation of CO impurities.^[^ ^13^, ^14^, ^15^, ^16^, ^17^ ^]^ However, it relies on precious metal Pt catalyst and is prone to CO poisoning, limiting activity and stability.^[^ ^18^, ^19^ ^]^ Alloying Bi with Pt alters electronic and surface properties, reducing CO poisoning and enhancing efficiency.^[^ ^20^, ^21^, ^22^, ^23^, ^24^, ^25^ ^]^ In addition, this strategy not only reduces the overall cost of the catalyst but also improves its performance.^[^ ^26^ ^]^

Despite recent advancements in rapid synthetic techniques such as Joule heating,^[^ ^27^ ^]^ pulsed electrochemical reduction,^[^ ^28^, ^29^ ^]^ and microwave heating^[^ ^30^, ^31^ ^]^ for alloy nanocatalysts, challenges persist in achieving long‐term stability, cost‐effectiveness, and catalytic activity.^[^ ^27^ ^]^ Femtosecond laser ablation in liquid provides a single‐step, surfactant‐free method for synthesizing metastable alloy nanoparticles with tunable compositions and defects under nonequilibrium conditions.^[^ ^32^, ^33^, ^34^ ^]^ This approach enables atomic‐level mixing, overcoming phase separation in conventional methods and introducing defects that enhance catalytic sites.

In this study, we synthesized platinum bismuth (PtBi) nanoalloys on a carbon support via femtosecond laser liquid ablation, achieving tunable compositions and exceptional catalytic properties. The optimized Pt_4_Bi/C catalyst exhibited remarkable performance in alkaline MOR, delivering a mass activity of 17.7 A mg^−1^_pt_ (11.5 times higher than 20% Pt/C) and a specific activity of 54.9 mA cm^−2^. *In‐situ* spectroscopy revealed a CO‐free reaction pathway enabled by Bi, effectively minimizing poisoning. The MOR–HER electrolyzer achieved 545 mV at 10 mA cm^−2^ and maintained stability for 54 h below 1.1 V, demonstrating potential for efficient hydrogen production. This work offers a new means for the general synthesis of high‐efficiency electrocatalysts for energy conversion and catalysis.

## 2 Results and Discussion

**Scheme 1:**
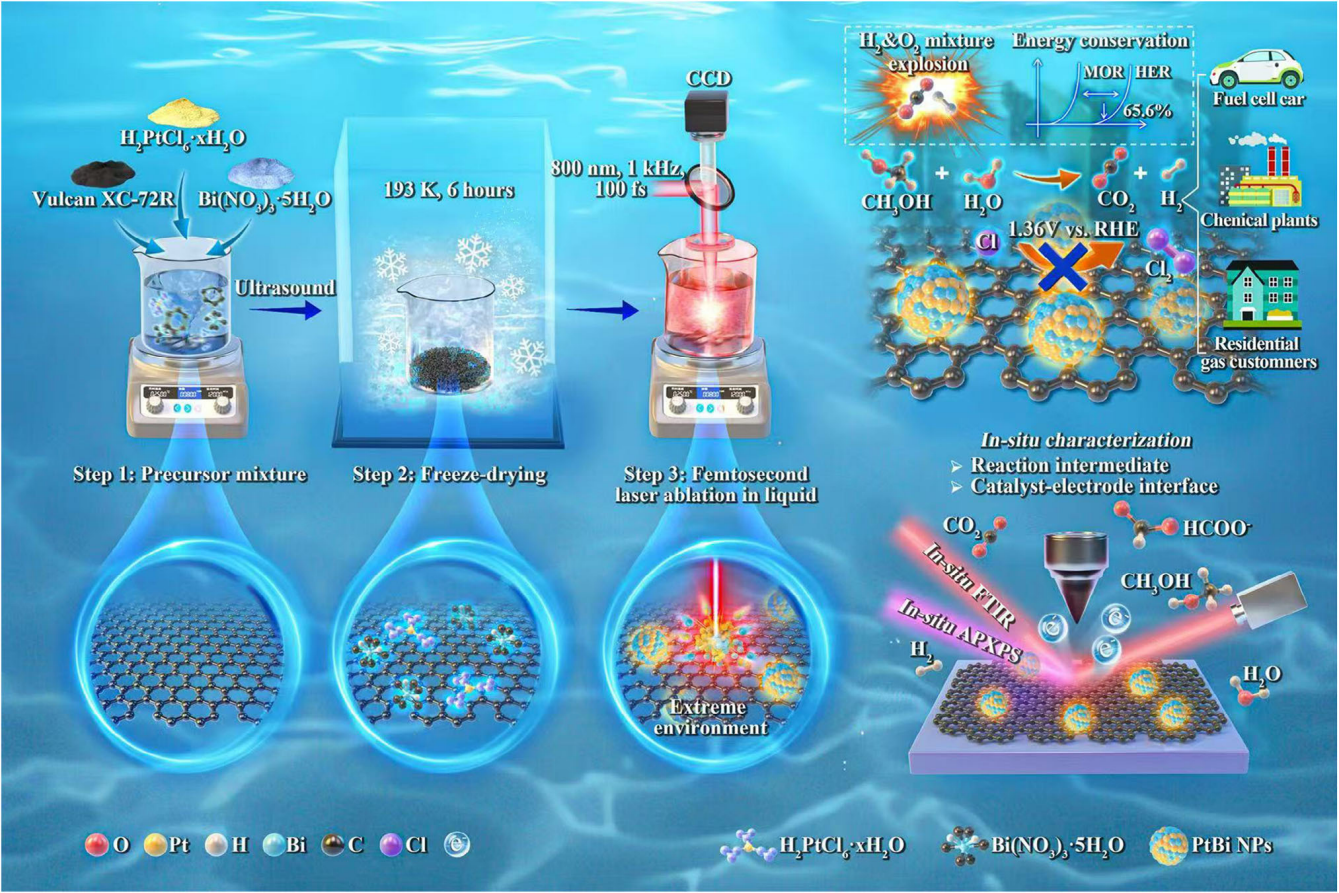
Schematic illustration of PtBi/C synthesis by femtosecond laser ablation in liquid, along with its catalytic applications in MOR‐assisted seawater electrolysis and *in*‐*situ* mechanistic studies.

The synthesis of PtBi catalysts supported on carbon (PtBi/C), the applications in MOR || HER seawater electrolysis and mechanistic studies are conceptually illustrated in **Scheme** **1**. The freeze‐dried powder was immersed in hexane and subjected to liquid‐phase ablation using a femtosecond laser (Figure S1, Supporting Information). This technique enabled nonequilibrium diffusion via ultrafast energy injection, facilitating the photoreduction of Pt^4+^/Bi^3+^ ions and resulting in the formation of metastable PtBi alloys, overcoming the limitations of traditional thermodynamic Pt─Bi phase diagrams, which typically require prolonged high‐temperature annealing for alloy synthesis.

**Figure 1:**
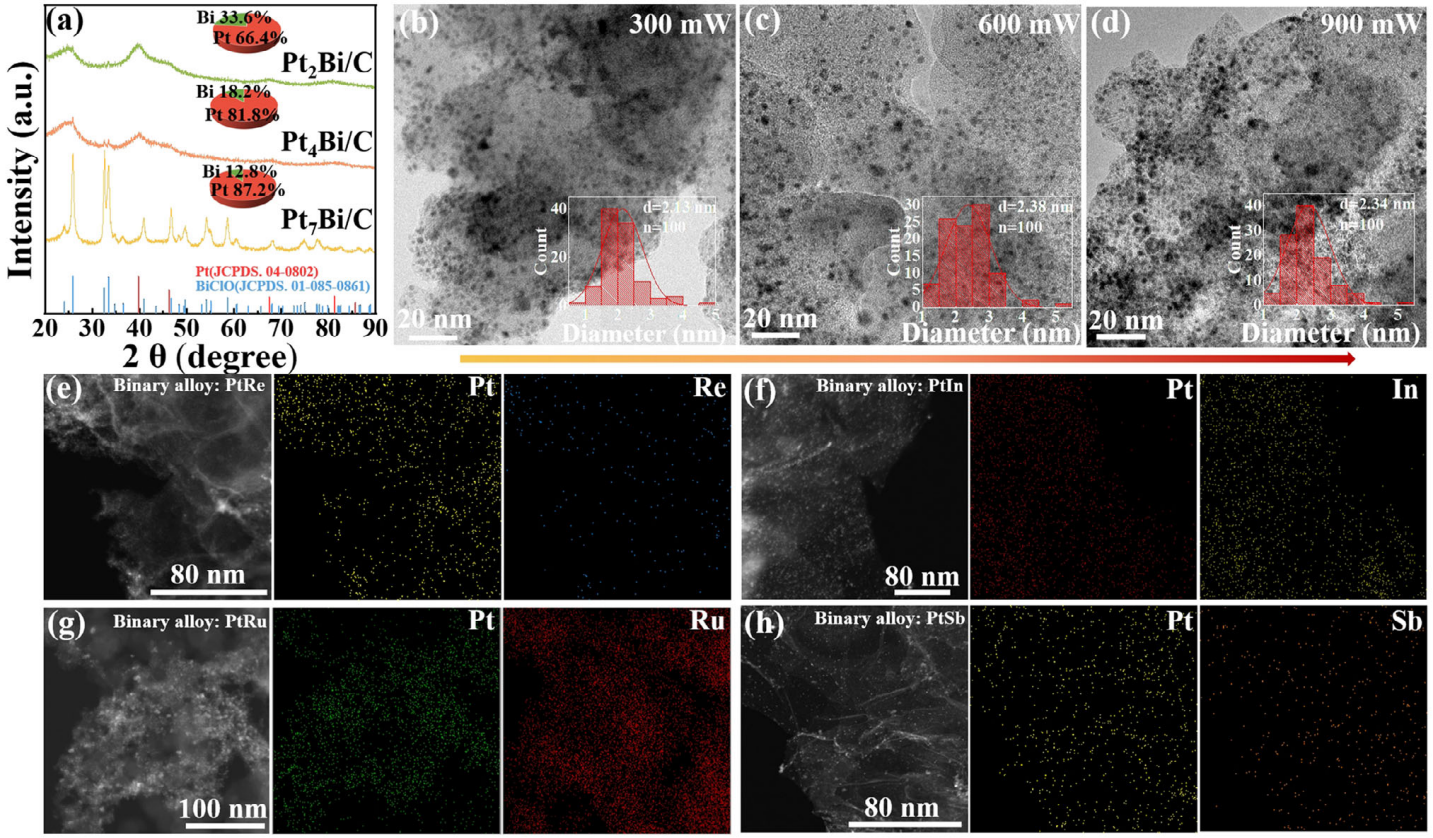
a) XRD patterns of Pt_7_Bi/C, Pt_4_Bi/C, and Pt_2_Bi/C. Red lines = Pt (JCPDS 04‐0802), blue lines = BiClO (JCPDS 01‐085‐0861). b–d) Transmission electron microscopy (TEM) images of Pt_7_Bi/C, Pt_4_Bi/C, and Pt_2_Bi/C. e–h) Scanning transmission electron microscopy–energy‐dispersive X‐ray spectroscopy (STEM–EDS) images of PtRe/C, PtIn/C, PtRu/C, and PtSb/C binary nanoalloys.

The influence of femtosecond laser power on the synthesis of PtBi nanoalloys was investigated systematically at 300, 600, and 900 mW. Inductively coupled plasma (ICP) analysis quantified Bi contents of 12.8% (300 mW), 18.2% (600 mW), and 33.6% (900 mW), corresponding to Pt:Bi molar ratios of 7:1 (Pt_7_Bi/C), 4:1 (Pt_4_Bi/C), and 2:1 (Pt_2_Bi/C), respectively (Table S1, Supporting Information). This compositional control arises from laser‐driven atomic redistribution, where femtosecond pulses selectively reduce precursors and regulate diffusion via nonthermal mechanisms, offering a robust method for tailoring PtBi catalyst properties. X‐ray diffraction (XRD) analysis indicates that the crystal structure of PtBi evolves with increasing laser power (**Figure** **1**a). At 300 mW, incomplete reduction results in the formation of transient Bi oxychloride (BiClO) phase (JCPDS 01‐085‐0861), which disappears at higher powers due to the formation of PtBi nanoalloys. Diffraction peaks correspond closely to the face‐centered cubic (fcc) structure of Pt (JCPDS 04‐0802), with a subtle shift attributed to Bi incorporation into the Pt lattice, confirming the formation of PtBi nanoalloys with an estimated average particle size of 2 nm, according to the Scherrer equation. No distinct diffraction features of Bi are observed, suggesting complete alloying of Bi with Pt into a uniform fcc framework. The slight shift (≈0.3°) in diffraction patterns as a function of increasing laser power indicates the incorporation of Bi into Pt lattice and the formation of PtBi nanoalloys.

Consistent with the XRD analysis, transmission electron microscopy (TEM) reveals that nanoparticle size remains consistently ≈2 nm across, as shown in Figure 1b–d and Figures S2–S5 (Supporting Information), underscoring the efficacy of femtosecond laser synthesis in preventing thermal aggregation. The ultrashort pulse duration, shorter than the electron–lattice relaxation time, limits energy dissipation to nonthermal electronic excitation, suppressing atomic migration and maintaining small, uniform particle sizes. Energy‐dispersive X‐ray spectroscopy (EDS) mapping confirms uniform precursor reduction and distribution on carbon supports.

While the present study primarily focuses on the synthesis and characterization of PtBi nanoalloys, this synthetic method was successfully extended to bimetallic systems to demonstrate its versatility, including PtIn (fcc–hcp), PtSb (fcc–rhombohedral), PtRu (fcc–hcp), and PtRe (fcc–hcp) (Figure 1e–h). These systems are challenging to synthesize using conventional methods due to thermodynamic constraints. For elements with high reduction potentials (Re^5+^), strict reduction requirements (Sb^3+^), or differing crystal structures (Ru and In), the femtosecond laser promotes efficient reduction and nonequilibrium solid solution formation through nonthermal electronic excitation and transient pressure fields. This spatiotemporal energy control bypasses thermodynamic equilibrium limitations, offering a versatile platform for designing multimetallic catalysts. TEM images of PtRu and PtRe (Figures S6 and S7, Supporting Information), confirm the formation of small, uniform nanoparticles, further illustrating the versatility of the femtosecond laser ablation technique.

**Figure 2:**
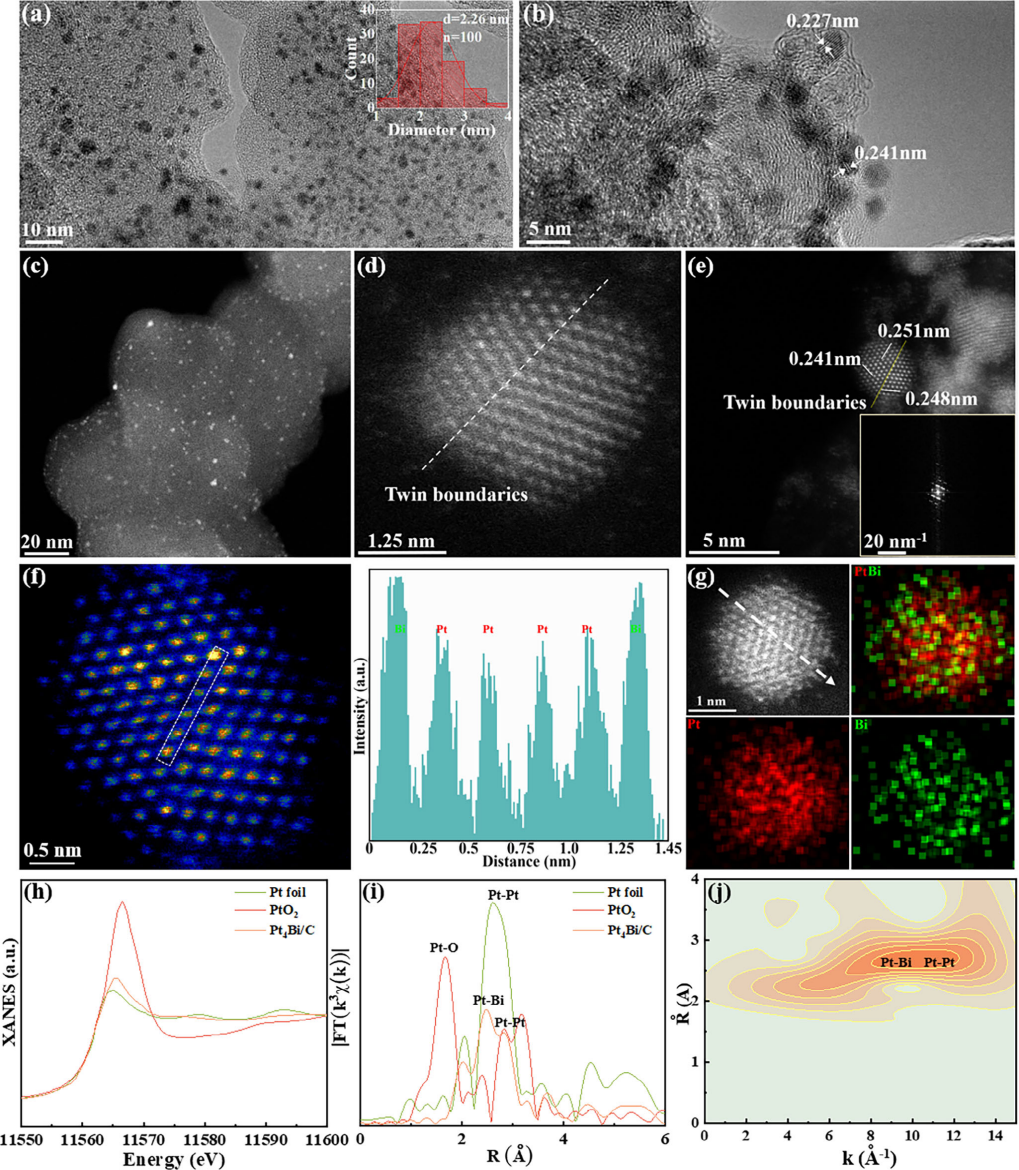
Structural and compositional characterization of Pt_4_Bi/C. a) TEM images and b) HR‐TEM image of Pt_4_Bi/C, with the inset histogram showing the size distribution of nanoparticles. c) Low‐magnification and d,e) High‐resolution HAADF‐STEM images of Pt_4_Bi/C. f) HAADF‐STEM image and the intensity profiles taken along the lines. g) STEM–EDS elemental mapping of Pt_4_Bi/C. h) X‐ray absorption near‐edge structure (XANES) spectra of Pt_4_Bi/C at the Pt L_3_‐edge, with Pt foil and PtO_2_ as references. i) Fourier transform and j) wavelet transform of the Pt L_3_‐edge extended X‐ray absorption fine structure (EXAFS) of Pt_4_Bi/C with *k*_3_ weighting.

High‐resolution‐TEM (HR‐TEM) reveals that PtBi nanoalloys are uniformly dispersed on carbon support (XC‐72R). The structural integrity of the carbon support remains intact following femtosecond laser ablation in liquid, as shown in **Figure** **2**a,b. High‐angle annular dark‐field scanning transmission electron microscopy (HAADF‐STEM) confirms the absence of nanoparticle agglomeration (Figure 2c). Twin boundaries are prevalent within the nanoparticles (Figure 2d,e), suggesting nonequilibrium crystallization, likely driven by rapid reduction processes and the coalescence of adjacent atom clusters under intense laser fields. HAADF‐STEM image shows isolated Bi atoms appearing brighter than Pt due to their higher atomic number, which enhances electron scattering. This effect is emphasized in HAADF‐STEM *Z*‐contrast imaging (Figure 2f). STEM–EDS mapping (Figure 2g) verifies the homogeneous distribution of Pt and Bi within the nanoalloy, with semiquantitative analysis indicating a Pt:Bi atomic ratio of ≈9:1 (Figure S8a, Supporting Information), supported by line‐scan profiles (Figure S8b, Supporting Information), confirming the formation of nanoalloys. The femtosecond laser synthesis method enables nonthermal atomic diffusion, bypassing the limitations of conventional thermodynamic phase diagrams.

To examine the local atomic and electronic structures of Pt_4_Bi/C, X‐ray absorption fine structure (XAFS) measurements were performed. The Pt L_3_‐edge X‐ray absorption near‐edge structure (XANES) analysis (Figure 2h) shows that the absorption edge and white line intensity for Pt_4_Bi/C fall between those of Pt foil and PtO_2_, with greater resemblance to Pt foil, indicating that the oxidation state of Pt in Pt_4_Bi/C is located between 0 and +4 and is closer to the metal valence state. Extended X‐ray absorption fine structure (EXAFS) analysis (Figure 2i) reveals a prominent peak at 2.6 Å, corresponding to Pt─Bi bonds. A weak peak at 2.9 Å, resembling the Pt foil signal, is attributed to Pt─Pt bond scattering. The strong Pt─Bi peak indicates alloying between Pt and Bi, suggesting that Bi atoms modify Pt's local coordination environment. Meanwhile, the weaker Pt─Pt peak points to diluted Pt─Pt interactions. High‐resolution wavelet transform (WT)‐EXAFS at the Pt L_3_‐edge was employed to examine the atomic configuration of Pt_4_Bi/C (Figure 2j). WT‐EXAFS improves the resolution of scattering contributions in *k*‐space and *R*‐space, allowing clearer distinction of overlapping coordination shells, especially in complex bimetallic systems like Pt_4_Bi/C. The WT contour plots reveal two intensity maxima: one at 9 Å^−1^ corresponding to the Pt─Bi pair and another at 11.2 Å^−1^ for Pt─Pt bonds, albeit with reduced intensity, confirming the bimetallic coordination structure of Pt_4_Bi/C. These distinct maxima highlight the coexistence of Pt─Bi and Pt─Pt bonds, with the reduced Pt─Pt intensity further corroborating the alloying effect that disrupts the metallic Pt lattice, consistent with a well‐dispersed Pt─Bi structure. EXAFS data at the Pt L_3_ edge were fitted using least‐squares methods, accounting for Pt─C, Pt─Pt, and Pt─Bi scattering paths. The analysis (Figure S9 and Table S2, Supporting Information) reveals a Pt─Bi bond length of 2.66 Å with a coordination number (CN) of 3.6 in the first shell and a Pt─Pt bond length of 2.78 Å (CN = 3.3) in the second shell. The fitting process provides quantitative structural parameters, where the higher CN of Pt─Bi compared to Pt─Pt suggests a preference for Pt coordination with Bi over Pt, indicative of a Pt─Bi alloy phase. Overall, the XAFS results underscore that Pt in Pt_4_Bi/C exists predominantly in a metallic state, with a local structure dominated by Pt─Bi interactions. This configuration likely contributes to its catalytic efficacy by optimizing the electronic.

X‐ray photoelectron spectroscopy (XPS) reveals that laser power affects the surface chemistry of PtBi nanoparticles. Survey spectra confirm the presence of Pt, Bi, C, and O in Pt_7_Bi/C, Pt_4_Bi/C, and Pt_2_Bi/C, indicating successful incorporation and oxidation of surface Pt and Bi under varied laser conditions (Figure S10, Supporting Information). Pt_7_Bi/C (300 mW) shows both metallic Pt^0^ and oxidized Pt^2+^, whereas Pt_4_Bi/C (600 mW) and Pt_2_Bi/C (900 mW) exhibit predominantly higher binding energy states (Figure S11a, Supporting Information). The metallic Pt fraction decreases from 61% at 300 mW to 28% at 600 mW, stabilizing at 23% for 900 mW. For Bi 4f (Figure S11b, Supporting Information), both Bi^0^ and Bi^3+^ are present, with Bi^3+^ prevailing at higher laser powers. This power‐dependent trend in oxidation states is noteworthy, as higher laser powers might intuitively be expected to promote greater reduction due to enhanced energy input. However, in femtosecond laser ablation in hexane, the process involves a delicate balance between reduction and oxidation. At elevated powers, stronger plasma and cavitation effects generate reactive oxygen species from trace sources (e.g., dissolved O_2_, residual impurities such as water, or carbon support functional groups), leading to postreduction oxidation of Pt and Bi surfaces. This results in decreased metallic Pt fractions (from 61% at 300 mW to 28% at 600 mW and 23% at 900 mW) and increased Bi^3+^ dominance, as observed. Despite this, the moderate oxidation in Pt_4_Bi/C (600 mW) optimizes the bifunctional role of Bi, enhancing MOR performance. Notably, XPS‐measured near‐surface Bi content (23.12% for Pt_4_Bi/C) exceeds bulk values from ICP‐OES (18.2%) and STEM–EDS (10.57%) (Table S1, Supporting Information), indicating a slight surface enrichment of Bi oxides.^[^ ^35^ ^]^

**Figure 3:**
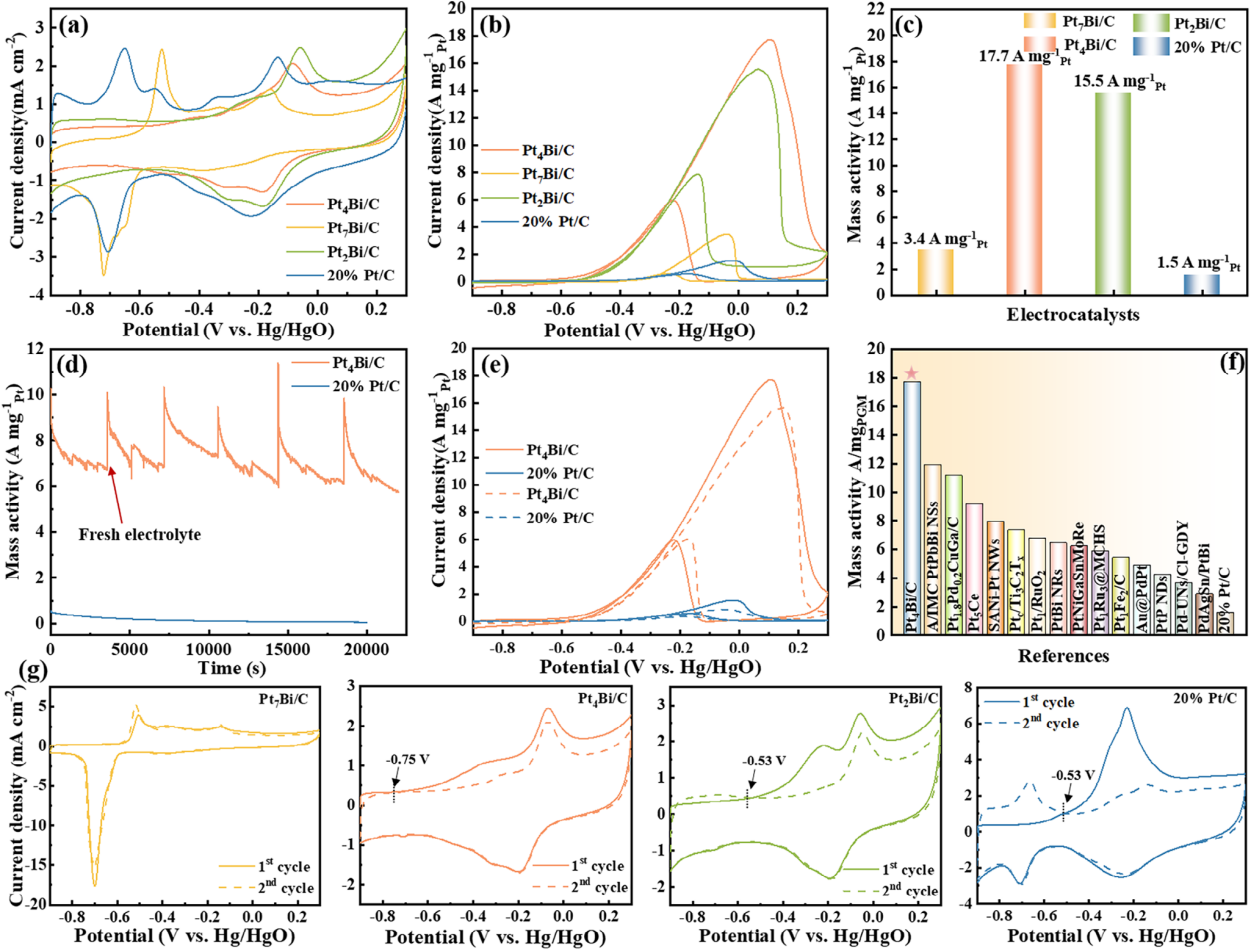
a) CV curves of various electrocatalysts in N_2_‐saturated 1.0 m KOH at 50 mV s^−1^. b) Mass‐normalized CV curves of various electrocatalysts in N_2_‐saturated 1 m KOH + 1 m CH_3_OH at 50 mV s^−1^. c) Mass activity histogram of various electrocatalysts. d) Chronoamperometry (CA) test of various electrocatalysts at −0.15 V. e) CV curves of 20% Pt/C and Pt_4_Bi/C in 1 m KOH + 1 m CH_3_OH before and after chronoamperometry test at 50 mV s^−1^. f) Comparison of mass activities between 20% Pt/C, Pt_4_Bi/C, and representative high‐performance MOR electrocatalysts in alkaline electrolytes. g) CO‐stripping curves of Pt_7_Bi/C, Pt_4_Bi/C, Pt_2_Bi/C, and 20% Pt/C.

**Figure** **3**a shows the cyclic voltammetry (CV) profiles of various catalysts in N_2_‐saturated 1 m KOH at 50 mV s^−1^. Prior to testing, the catalysts were activated until stable CV curves were achieved. The CV curves are categorized into two regions: the hydrogen adsorption/desorption region (*E* < −0.47 V) and the OH adsorption/desorption (OH_ads/des_) region (*E* > −0.37 V, including −0.2 to 0.1 V). Compared to 20% Pt/C, PtBi showed no hydrogen adsorption/desorption peaks, which is attributed to the modification of the Pt surface by Bi (oxides).^[^ ^36^ ^]^ For Pt_7_Bi/C, an oxidation peak at −0.52 V corresponds to the oxidation of Bi sites, while a reduction peak at −0.72 V is due to the reduction of BiO_2_^−^ species.^[^ ^37^ ^]^ However, these peaks are absent in Pt_4_Bi/C and Pt_2_Bi/C, further revealing that Bi (oxide) is atomically dispersed with Pt without phase segregation. In the OH_ads/des_ region, the incorporation of Bi leads to a positive shift in the oxidation peaks of Pt_4_Bi/C (≈−0.08 V) and Pt_2_Bi/C (≈−0.06 V) compared to 20% Pt/C (≈−0.13 V). This positive shift indicates weakened OH adsorption on Pt, which is due to Bi incorporation downshifting the Pt d‐band center. Notably, as Bi content increases, the OH peak shifts more positively, reflecting stronger electronic modulation by Bi that enhances MOR efficiency by promoting intermediate desorption and reducing site blocking.^[^ ^38^ ^]^

Pt_4_Bi/C demonstrated a mass activity of 17.7 A mg^−1^_Pt_ in alkaline MOR (Figure 3b,c), surpassing Pt_2_Bi/C (15.5 A mg^−1^_Pt_), Pt_7_Bi/C (3.4 A mg^−1^_Pt_), and 20% Pt/C (1.5 A mg^−1^_Pt_) by factors of 1.1, 5.1, and 11.5, respectively. This performance ranks Pt_4_Bi/C among the best‐reported mass activities for MOR in alkaline electrolytes (Figure 3f and Table S3 (Supporting Information)).^[^ ^14^, ^39^, ^40^, ^41^, ^42^, ^43^, ^44^, ^45^, ^46^, ^47^, ^48^ ^]^ Additionally, Pt_4_Bi/C exhibited a more negative onset potential than 20% Pt/C, indicating a lower energy barrier for MOR initiation. Electrochemical surface area (ECSA) measurements using copper underpotential deposition showed values of 18.63 m^2^ g^−1^ for Pt_4_Bi/C and 43.68 m^2^ g^−1^ for 20% Pt/C (Figure S12, Supporting Information). Despite its smaller ECSA, Pt_4_Bi/C achieved a specific activity of 54.9 mA cm^−2^ (Figure S10, Supporting Information), which is 15.6 times higher than that of 20% Pt/C (3.5 mA cm^−2^). This improvement reflects an increase in the intrinsic activity per active site, outperforming previously reported Pt‐based and other electrocatalysts (Table S3, Supporting Information).

The durability of the catalysts was evaluated using chronoamperometry (CA) in N_2_‐saturated 1 m KOH + 1 m CH_3_OH at −0.15 V. As shown in Figure 3d, 20% Pt/C exhibited rapid activity decay due to poisoning by *CO intermediates, whereas Pt_4_Bi/C showed a slower decay rate and maintained stable performance over 21990 s. Notably, the catalytic activity of Pt_4_Bi/C can be restored completely by replacing fresh working electrolytes after six consecutive cycles of CA tests, revealing respectable reproducibility. Furthermore, Pt_4_Bi/C exhibited structural stability in alkaline electrolytes, with the average size and nanoparticle morphology remaining intact after CA testing (Figures S14 and S15, Supporting Information). This structural stability is attributed to the metastable structure formed through the nonequilibrium process by the femtosecond laser ablation technique. This process promotes atomic‐level dispersion of Pt and Bi, effectively preventing elemental segregation. Moreover, the carbon carrier after laser treatment maintains its structural integrity, providing stable physical support for the nanoparticles, thereby further enhancing the durability of the catalyst. The CV curve of Pt_4_Bi/C was insignificant after the CA test, and the mass activity decreased by 11.2% (Figure 3e). CO desorption curves revealed that the oxidation peak of adsorbed CO on Pt_4_Bi/C was significantly suppressed at ≈−0.23 V compared to 20% Pt/C, suggesting enhanced tolerance to CO poisoning due to the Bi‐modified surface (Figure 3g).^[^ ^49^ ^]^ Specifically, 20% Pt/C shows an intense CO oxidation peak spanning −0.4 to 0.2 V, indicating severe *CO poisoning from strong CO adsorption. In contrast, Pt_4_Bi/C exhibits a reduced peak area and ≈0.17 V delayed onset, proving negligible *CO adsorption. This is enabled by Bi promoting a CO‐free pathway, as confirmed by *in*‐*situ* Fourier transform infrared spectroscopy (FTIR) (rapid HCOO^−^ oxidation without CO) and density functional theory (DFT) (lower *CO adsorption energy on PtBi─O). A reasonable concentration of Bi on the surface promotes a CO‐free reaction pathway, thereby ensuring superior activity and durability for MOR in alkaline electrolytes.

While the Pt_4_Bi/C catalyst is optimized for alkaline MOR, its performance in acidic media was also evaluated for completeness. As shown in Figure S16a (Supporting Information), CV curves of Pt_4_Bi/C and commercial Pt/C were recorded in N_2_‐saturated 0.5 m H_2_SO_4_ at a scan rate of 50 mV s^−1^. In 0.5 m H_2_SO_4_ + 1 m CH_3_OH (Figure S16b, Supporting Information), Pt_4_Bi/C exhibits a mass activity of 2.06 A mg^−1^ Pt, which is 4.6 times higher than that of commercial Pt/C (0.45 A mg^−1^ Pt). Chronoamperometry curves at 0.6 V (vs Ag/AgCl) in the same electrolyte (Figure S16c, Supporting Information) demonstrate that Pt_4_Bi/C maintains superior stability, with a slower current decay over 5000 s compared to Pt/C, attributed to reduced poisoning by carbonaceous intermediates. This performance is competitive with or superior to reported Pt‐based catalysts in acidic MOR (Table S4, Supporting Information), although lower than in alkaline conditions due to the less favorable role of Bi in proton‐rich environments.

**Figure 4:**
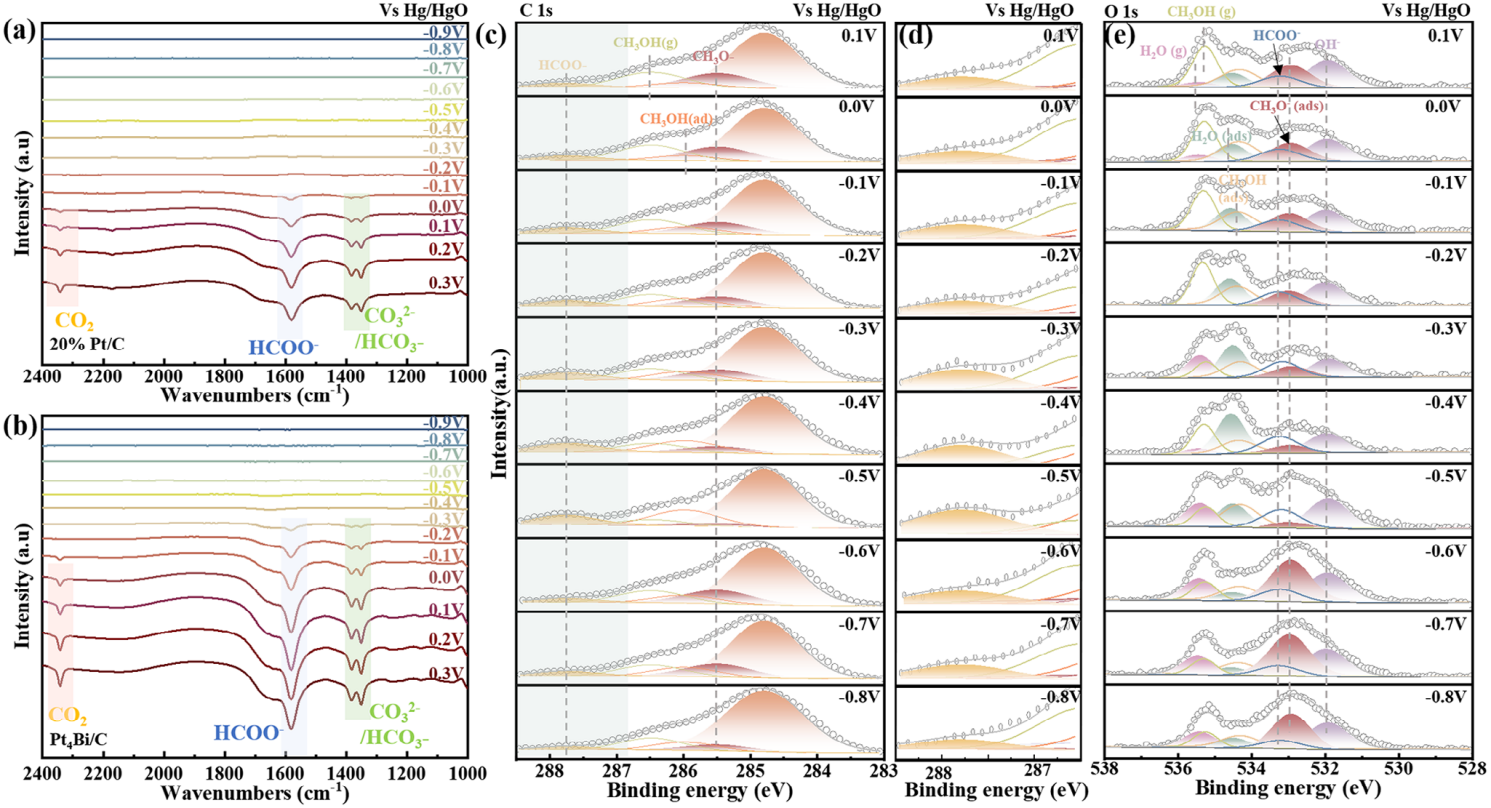
*In*‐*situ* FTIR spectra (1000–2400 cm^−1^) of a) 20% Pt/C and b) Pt_4_Bi/C were recorded in a N_2_‐saturated 1 m KOH + 1 m CH_3_OH. c,d) Ambient pressure X‐ray photoelectron spectroscopy (APXPS) photoemission spectra of C 1s lines for the Pt_4_Bi/C catalyst surface under operational conditions. e) APXPS photoemission spectra of O 1s lines for the Pt_4_Bi/C catalyst surface under operational conditions. All photoemission spectra were calibrated with adventitious hydrocarbons at 284.8 eV and were corrected using a Shirley background. C 1s spectra were deconvoluted into the following components: sp^3^ carbon (284.8 eV), CH_3_O^−^ (≈285.5 eV), CH_3_OH (ads, ≈286 eV), CH_3_OH (g, ≈286.5 eV), and HCOO^−^ (≈287.8 eV). O 1s spectra were deconvoluted into the following contributions: OH^−^ (≈531.9 eV), CH_3_O^−^ (ads, ≈532.9 eV), HCOO^−^ (≈533.2 eV), CH_3_OH (ads) (≈534.3 eV), H_2_O (ads, ≈534.5 eV), CH_3_OH (g, ≈535.2 eV), and H_2_O (g, ≈535.4 eV).

*In*‐*situ* FTIR spectroscopy was employed to investigate the reaction pathways and intermediates of MOR catalyzed by Pt_4_Bi/C and 20% Pt/C (**Figure** **4**a,b), offering insights into their reaction mechanism in alkaline media. The spectra revealed several distinct vibrational bands that track the evolution of intermediates and products. For both catalysts, the band at ≈1585 cm^−1^, attributed to the asymmetric stretching vibration of HCOO^−^, emerges as a key indicator of methanol oxidation. This suggests an initial dehydrogenation and C─O bond cleavage pathway, forming HCOO^−^ as an intermediate, consistent with the formic acid route. As the electrode potential increases, the behavior of these HCOO^−^ bands diverges between Pt_4_Bi/C and 20% Pt/C, reflecting the influence of Bi doping. On Pt_4_Bi/C, the HCOO^−^ peak at 1585 cm^−1^ appears at a lower potential and intensifies more rapidly than on 20% Pt/C, indicating that Bi enhances the adsorption and activation of CH_3_OH, likely due to electronic modifications of Pt sites. This intermediate is subsequently oxidized to CO_2_, as evidenced by the vibrational band at ≈2345 cm^−1^. Remarkably, Pt_4_Bi/C initiates CO_2_ production 200 mV earlier than 20% Pt/C, underscoring a reduced energy barrier for the complete oxidation pathway. At higher potentials, the adsorbed CO_2_ desorbs and reacts with OH^−^ to form CO_3_^2−^/HCO_3_^−^, marked by a broad vibrational band at 1376 cm^−1^, observed consistently for both catalysts. In the absence of Bi, 20% Pt/C shows a slower progression through these reaction steps, emphasizing Bi's role in optimizing the reaction dynamics of MOR, possibly by enhancing local OH^−^ availability or modifying Pt's electronic properties. This comparison elucidates the critical contribution of alloyed Bi (oxide) to enhancing catalytic efficiency. By facilitating the formation of kinetically relevant intermediates, lowering energy barriers, and accelerating their conversion to CO_2_, Bi alloying in Pt_4_Bi/C markedly improves MOR kinetics compared to 20% Pt/C. The broad peak in the range of 3200–3600 cm^−1^ and the peak at ≈1621 cm^−1^ can be assigned to the stretching (O─H) mode and the bending δ(H─O─H) mode of interfacial water molecules (Figure S17, Supporting Information), respectively. At −0.3 V, Pt_4_Bi/C exhibits a distinct OH^−^ vibration peak, whereas no detectable signal is observed for 20% Pt/C. As the potential increases, the OH^−^ peak intensity on Pt_4_Bi/C further rises, indicating that Bi facilitates stronger and more stable OH^−^ adsorption, thereby enhancing local OH^−^ concentration and accelerating the oxidation of MOR intermediates.

*In*‐*situ* dynamic monitoring of MOR in alkaline media was conducted using synchrotron‐based APXPS with a custom‐designed three‐electrode reaction cell (Figure S18, Supporting Information). Reaction intermediates were identified through spectral analysis of the C 1s and O 1s from −0.8 to 0.1 V (Figure 4c,d and Figure S20 (Supporting Information)). The C 1s spectra exhibited five characteristic peaks, including sp^3^ carbon (≈284.8 eV), CH_3_O^−^ (285.5 eV), CH_3_OH (ads) (≈286 eV), CH_3_OH (g) (≈286.5 eV), and HCOO^−^ (≈287.8 eV), indicating C─O(H) bonds and methanol chemisorption (Figure 4c).^[^ ^50^, ^51^ ^]^ The O 1s spectra (Figure 4e) revealed that the catalyst surface was primarily covered by H_2_O (g), CH_3_OH (g), and OH^−^ species and the peaks at 534.3 and 534.5 eV correspond to CH_3_OH (ads) and adsorbed H_2_O, respectively. When a potential of −0.5 V was applied, the C 1s spectrum exhibited a peak at 287.8 eV, indicating the formation of HCOO^−^ intermediates. This was further confirmed by the zoom‐in view of C 1s (Figure 4d) and O 1s spectrum with a peak at 533.2 eV attributed to HCOO^−^. At potentials above −0.5 V, the HCOO^−^ signal in both C 1s and O 1s spectra decreased with increasing potential, which can be attributed to its further oxidation to CO_2_. The evolution of HCOO^−^ manifests its central role in the reaction coordinates of MOR. Additionally, the decreased intensity of the CH_3_O^−^ peak at 285.5 eV in the C 1s spectrum suggests the generation of methoxide from methanol dehydrogenation. OH^−^ plays a crucial role in MOR by promoting CH_3_O^−^ formation through nucleophilic attack on the O─H bond in CH_3_OH. As the coverage of CH_3_OH diminished, the presence of kinetically relevant species, such as CHO^−^ and HCOO^−^, on the Pt_4_Bi/C surface became more evident, underscoring their importance in MOR. Furthermore, the significant reduction in OH^−^ at 531.9 eV at −0.4 V suggests its active participation in oxidizing HCOO^−^. To quantitatively track the evolution of these intermediates under varying reaction potentials, we calculated their relative contents based on the deconvoluted peak areas in the C 1s and O 1s spectra and plotted them against applied potential (Figure S19, Supporting Information). At potentials below −0.6 V, the CH_3_O^−^ content is high, reflecting initial methanol dehydrogenation. As the potential shifts to −0.5 V and above, a pronounced increase in HCOO^−^ is observed, paralleled by a decline in CH_3_O^−^ and OH^−^, indicating Bi‐promoted OH^−^ incorporation for oxidizing CH_3_O^−^ to HCOO^−^. The subsequent rapid decay of HCOO^−^ at higher potentials confirms its efficient transformation to CO_2_, underscoring the lowered energy barrier facilitated by Bi alloying. These quantitative dynamics reinforce the proposed formic acid pathway, highlighting Bi's electronic modulation that enhances intermediate adsorption/desorption balance. *In*‐*situ* FTIR and APXPS results collectively demonstrate that the MOR on Pt_4_Bi/C proceeds via a formic acid pathway, with HCOO^−^ serving as a key intermediate that is further oxidized to CO_2_ at lower potentials compared to pure Pt catalysts.

**Figure 5:**
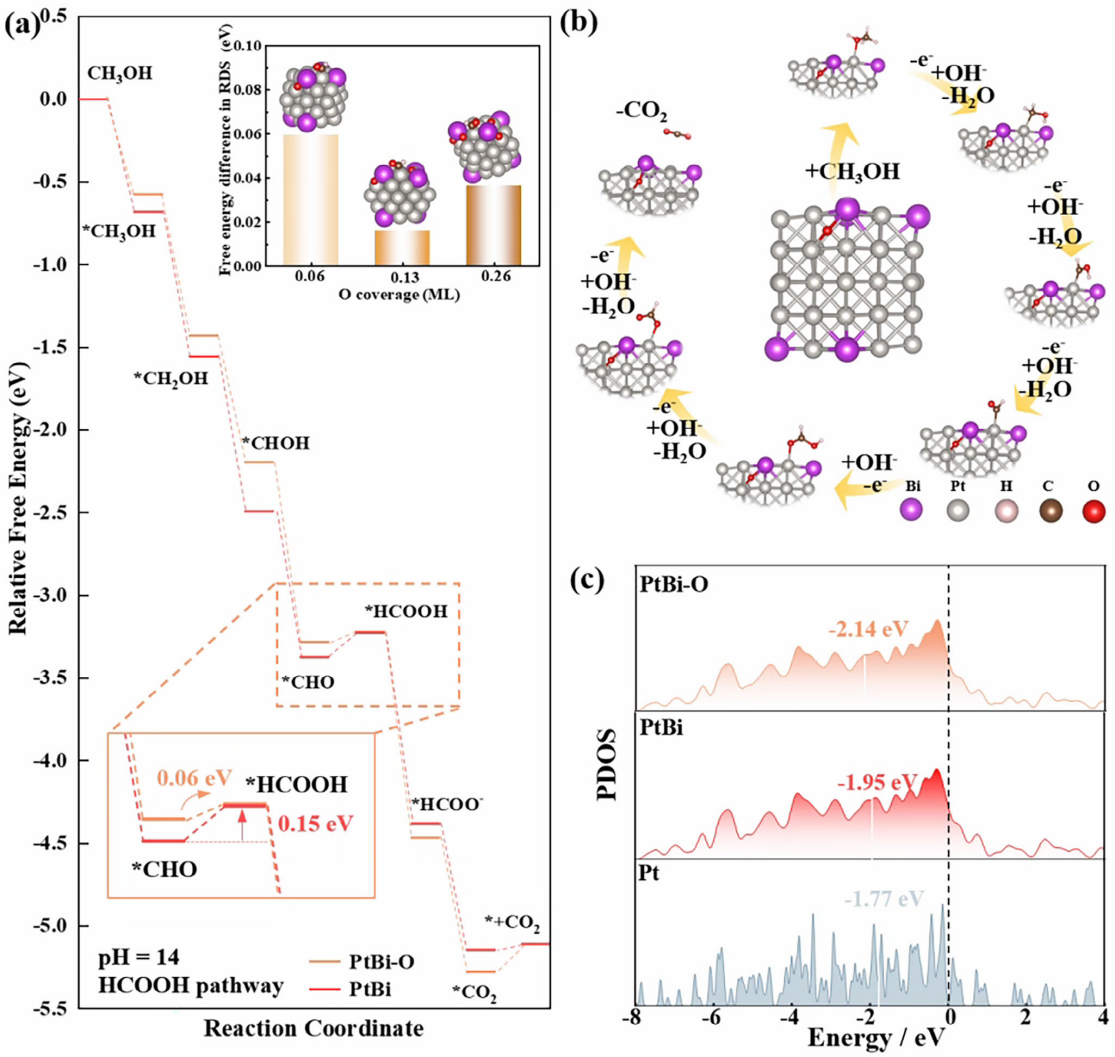
a) Free‐energy profiles of MOR on PtBi and PtBi─O clusters. Oxygen atom coverage is estimated based on the ratio of oxygen atoms to the total number of metal atoms exposed on the top half of the nanocluster. b) Space‐filling models of the optimized structure showing the CO_2_ pathway of the MOR. c) PDOS of the Pt d‐orbital in Pt, PtBi, and PtBi─O clusters.

To elucidate the impact of Bi doping on PtBi, we constructed models of both PtBi and oxygenated PtBi─O and performed DFT calculations (Figures S20 and S21, Supporting Information). Note that the oxidation of Bi is anticipated in both femtosecond laser ablation and the working conditions of MOR, as indicated in our XPS analysis. The enhanced MOR kinetics of PtBi are attributed to modifications in the electronic structure. We investigated this effect by calculating the projected density of states (PDOS) for the d‐orbitals of Pt, PtBi, and PtBi─O clusters. The potential‐determining step for Pt, PtBi, and PtBi─O is the oxidation of *CHO to *HCOOH. PtBi─O exhibits an energy barrier lower than that of Pt and PtBi, indicating that the introduced oxidation effect of PtBi accelerates the kinetics of *CHO conversion to *HCOOH (**Figure** **5**a). As depicted in the inset of Figure 5a, variations in oxygen atom coverage influence the free energy barrier of the RDS, specifically the oxidation of *CHO to *HCOOH. Specifically, an increase in oxygen atom coverage from 0.06 to 0.13 lowers the free energy barrier, suggesting that a moderate oxygen level optimizes the PtBi electronic structure, lowering the activation energy and enhancing *CHO to *HCOOH conversion. However, further increases in oxygen atom coverage from 0.13 to 0.26 raise the free energy barrier, likely due to excessive modification of the PtBi surface's electronic structure. This rise is attributed to surplus oxygen overly altering the PtBi electronic structure, weakening the binding of intermediates and hindering the reaction. This nonlinear trend underscores the dual role of oxygen coverage in the catalytic process: moderate oxidation enhances catalytic activity, whereas excessive coverage may induce negative effects. Additionally, adsorption energy calculations confirmed that PtBi─O reduces the adsorption of poisoning intermediates such as *CO and strengthens the adsorption of *OH (Figure S22, Supporting Information), alleviating the poisoning of Pt‐based electrode and promoting the reaction kinetics of MOR. It ensures the efficient and stable performance of Pt_4_Bi/C in long‐term electrochemical reactions. The adsorbed CH_3_OH molecules reacting with surface‐adsorbed *OH species and eventually converted into CO_2_ follow the sequence of CH_3_OH →*CH_3_OH → *CH_2_OH → *CHOH → *CHO → *HCOOH → HCOO^−^ → *CO_2_ → *+CO_2_ (Figure 5b). As shown in Figure 5c, the d‐band center of Pt in PtBi clusters shifted to −1.95 eV compared to −1.77 eV in Pt clusters. However, upon introducing O, the d‐band center further shifted to −2.14 eV in PtBi─O. The proximity of the d‐band center to the Fermi energy level empirically correlates with the adsorption strength of the reactive intermediates.^[^ ^52^ ^]^ As the d‐band center shifts away from the Fermi level, intermediate adsorption weakens (CHO/CO) on Pt sites (DFT calculations show a 0.02 eV reduction in *CO adsorption energy and enhanced *OH binding, Figure S22 (Supporting Information)). This weakened adsorption of *CO reduces catalyst poisoning, while the optimized binding of *OH facilitates the oxidation of *CHO to *HCOOH, directly promoting the kinetics of the RDS and accelerating the overall MOR pathway. The shift in the d‐band center of Pt in PtBi─O, as calculated by DFT, is consistent with *in*‐*situ* FTIR evidence of CO‐free pathways and rapid HCOO^−^ oxidation, highlighting how Bi doping and oxidation optimize the Pt electronic environment for enhanced electrocatalytic efficiency. Collectively, *in*‐*situ* FTIR, APXPS, and DFT analyses demonstrate that the superior MOR performance of Pt_4_Bi/C arises from a Bi‐ and O‐modified electronic structure that accelerates HCOO^−^ oxidation and mitigates *CO adsorption (evidenced by lower adsorption energies in DFT), with experimental data indicating earlier CO_2_ production and theoretical insights revealing a reduced *CHO oxidation barrier, thus providing a comprehensive understanding of the catalytic synergy.

**Figure 6:**
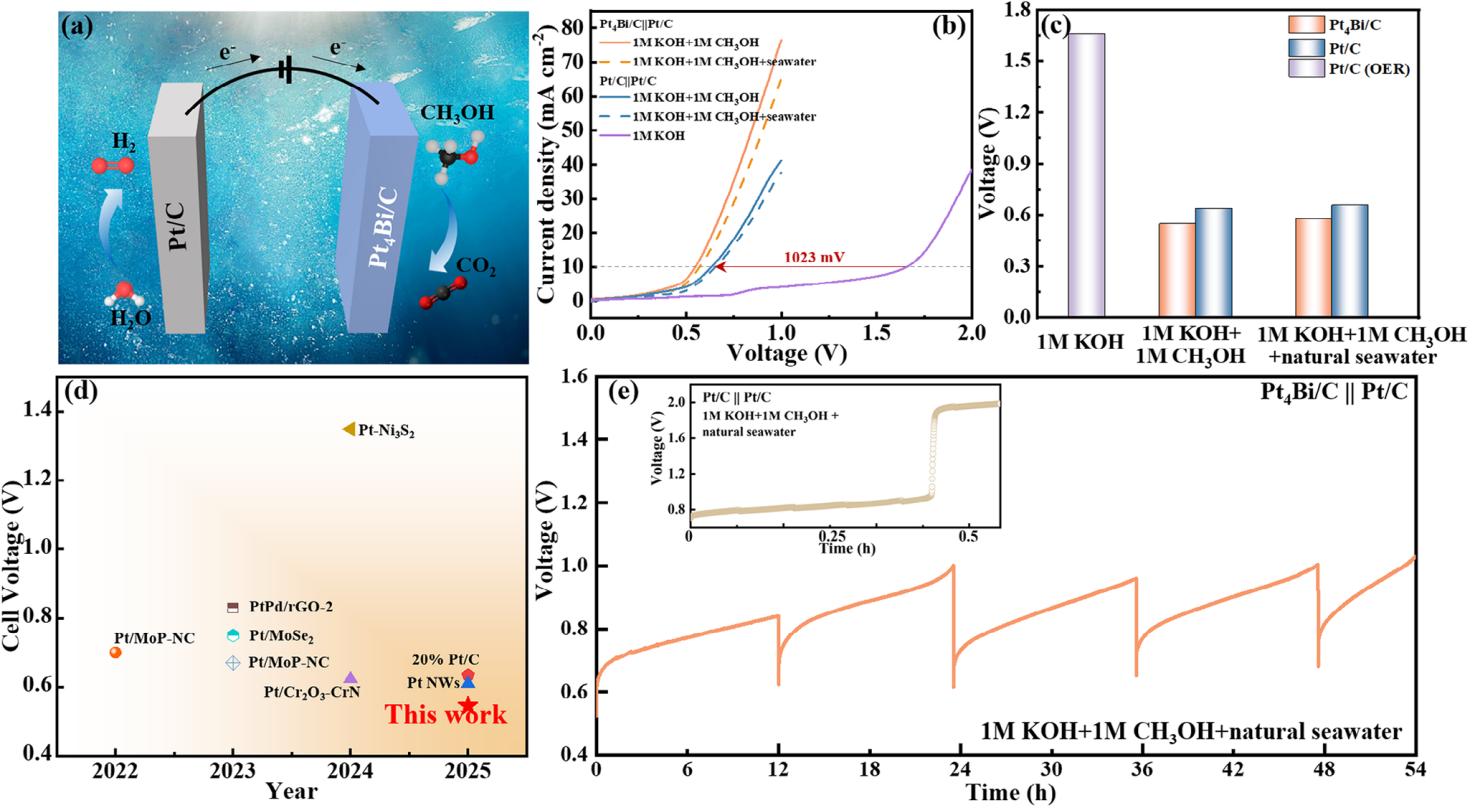
a) Schematic of the methanol‐water electrolyzer setup. b) LSV curves of the MOR‐assisted seawater electrolysis with different electrode pairs in various electrolytes at 50 mV s^−1^. Catalyst loadings: 0.01 mg cm^−2^ (Pt_4_Bi/C), 0.05 mg cm^−2^ (Pt/C). c) The voltage required for MOR‐assisted seawater electrolysis at 10 mA cm^−2^ in different electrolytes. d) Comparison of voltages at 10 mA cm^−2^ for the Pt_4_Bi/C catalyst in this study and previously reported catalysts. e) Chronopotentiometric curves of the MOR‐assisted seawater electrolysis at 10 mA cm^−2^ in alkaline natural seawater.

The effect of Cl^−^ ions on the seawater electrolytic performance of Pt/C || Pt_4_Bi/C and Pt/C || Pt/C pairs was investigated in 1 m KOH + 1 m CH_3_OH + seawater (**Figure** **6**a). The presence of Cl^−^ ions slightly decreases the overall activity of the cell, possibly due to repulsive force near the anode surface that hinders the adsorption of CH_3_OH (Figure 6b).^[^ ^53^ ^]^ In two‐electrode electrolysis, the geometric current density is regulated by the catalyst loading and mass transfer. Therefore, the limitation of methanol diffusion may affect the reaction kinetics. Additionally, replacing OER with MOR in the Pt/C || Pt/C system significantly lowers the voltage by 1023 to 633 mV at 10 mA cm^−2^, corresponding to a 61.7% decrease. Furthermore, using PtBi as the anode reduces the voltage by an additional 90 mV (Figure 6c). Mass transport differences arise from minimized diffusion limitations in three‐electrode setups compared to potential methanol concentration gradients in the two‐electrode electrolyzer, particularly in high‐ionic‐strength seawater‐like electrolytes. The rate‐determining step may shift from intrinsic surface reactions (*CHO to *HCOOH) in half‐cells to transport or Ohmic losses in full‐cells due to uncompensated resistance. Interfacial effects, including nonuniform potential distribution and bubble‐induced site blocking, further moderate the enhancement. Notably, the Pt_4_Bi/C || Pt/C electrolyzer achieved a voltage of 545 mV at 10 mA cm^−2^, outperforming all currently reported catalysts (Figure 6d and Table S5 (Supporting Information)).^[^ ^8^, ^54^, ^55^, ^56^ ^]^ The hydrogen production rate was ≈0.07 mL min^−1^ cm^−2^, with Faradaic efficiencies close to 100% at applied potentials of 0.6, 0.8, and 1.0 V (Figure S24, Supporting Information). These metrics demonstrate the system's efficiency as an initial proof of concept in complex media. This demonstrates the excellent potential of the Pt_4_Bi/C || Pt/C electrolyzer for hydrogen production. The stability of the seawater‐coupled electrolysis system was evaluated by conducting chronopotentiometry at 10 mA cm^−2^, with the electrolyte replaced every 12 h. Importantly, the Pt_4_Bi/C || Pt/C pair showed excellent durability, continuously generating H_2_ for 54 h at 10 mA cm^−2^ in natural seawater at voltages below 1.1 V with minimal degradation (Figure 6e), while the Pt/C || Pt/C electrolyzer failed after only 1600 s. The gradual increase in voltage may result from depletion of methanol and the accumulation of toxic intermediates on the catalyst surface.^[^ ^57^ ^]^ Furthermore, the Pt_4_Bi catalyst demonstrated superior performance compared to previously reported catalysts (Table S6, Supporting Information)

## 3 Conclusion

In summary, we successfully synthesized a series of ligand‐free PtBi catalysts using femtosecond laser liquid‐phase ablation. Mechanistic studies reveal that incorporating Bi, an oxophilic element, enhances the electrocatalytic efficiency of Pt‐based catalysts for MOR. This enhancement arises from a synergistic electronic–structural effect, the hybridization of the p‐orbital of Bi with the d‐orbital of Pt creates an electron‐transfer pathway, shifting the d‐band center of PtBi downward by 0.37 eV relative to pure Pt. Additionally, Bi introduction optimizes the surface reaction environment via a bifunctional mechanism. PtBi─O exhibits stronger *OH adsorption compared to pure Pt, thereby accelerating the removal of reaction intermediates such as ^*^CHO and ^*^HCOO^−^. In the MOR pathway, the *CHO to HCOOH step is rate limiting, and Bi doping reduces its energy barrier. The transient high temperature and high‐pressure conditions of the femtosecond laser enabled ligand‐free surface construction, addressing the issue of active site blocking by organic ligands in conventional synthesis methods. The mass activity (17.7 A mg^−1^_Pt_) is 11.5 times that of 20% Pt/C, with enhanced durability and resistance to poisoning. The Pt_4_Bi/C || Pt/C electrolyzer achieved a voltage of 545 mV at 10 mA cm^−2^, outperforming all currently reported catalysts. Furthermore, Pt_4_Bi/C operated successfully in natural seawater at 10 mA cm^−2^ for 54 h at voltages below 1.1 V. This demonstrates the feasibility of MOR‐assisted seawater electrolysis for hydrogen production and underscores the potential for energy‐efficient, chlorine‐free seawater electrolysis.

## Conflict of Interest

The authors declare no conflict of interest.

## Supporting information



Supporting Information

## Data Availability

The data that support the findings of this study are available from the corresponding author upon reasonable request.

## References

[1] J. Gu , L. Li , Q. Yang , F. Tian , W. Zhao , Y. Xie , J. Yu , A. Zhang , L. Zhang , H. Li , J. Zhong , J. Jiang , Y. Wang , J. Liu , J. Lu , J. Am. Chem. Soc. 2024, 146, 5355. 38358943 10.1021/jacs.3c12419

[2] Q. Sha , S. Wang , L. Yan , Y. Feng , Z. Zhang , S. Li , X. Guo , T. Li , H. Li , Z. Zhuang , D. Zhou , B. Liu , X. Sun , Nature 2025, 639, 360. 40044863 10.1038/s41586-025-08610-1

[3] J. Zhang , X. Fu , S. Kwon , K. Chen , X. Liu , J. Yang , H. Sun , Y. Wang , T. Uchiyama , Y. Uchimoto , S. Li , Y. Li , X. Fan , G. Chen , F. Xia , J. Wu , Y. Li , Q. Yue , L. Qiao , D. Su , H. Zhou , W. A. Goddard , Y. Kang , Science 2025, 387, 48. 39745949 10.1126/science.ado9938

[4] X. Kang , F. Yang , Z. Zhang , H. Liu , S. Ge , S. Hu , S. Li , Y. Luo , Q. Yu , Z. Liu , Q. Wang , W. Ren , C. Sun , H.‐M. Cheng , B. Liu , Nat. Commun. 2023, 14, 3607. 37330593 10.1038/s41467-023-39386-5 PMC10276855

[5] Y. Zhu , Y. Chen , Y. Feng , X. Meng , J. Xia , G. Zhang , Adv. Mater. 2024, 36, 2401694. 10.1002/adma.202401694 38721895

[6] S. Zhang , C. Zhang , X. Zheng , G. Su , H. Wang , M. Huang , Appl. Catal., B 2023, 324, 122207.

[7] A. Zhang , F. Yang , Y. Luo , X. Shen , Q. Yao , K. Wang , J. Xia , Z.‐H. Lu , Appl. Catal., B 2025, 373, 125360.

[8] K. Deng , X. Liu , P. Liu , X. Lv , W. Tian , J. Ji , Angew. Chem., Int. Ed. 2024, 64, 202416763. 10.1002/anie.202416763 39523460

[9] X. Deng , X. Kang , M. Li , K. Xiang , C. Wang , Z. Guo , J. Zhang , X.‐Z. Fu , J.‐L. Luo , J. Mater. Chem. A 2020, 8, 1138.

[10] X. Hu , I. U. Islam , B. Guo , J. Shang , X. Zhao , X. Wang , U. Farooq , Appl. Catal., B 2025, 376, 125431.

[11] Y. Yang , X. Wu , M. Ahmad , F. Si , S. Chen , C. Liu , Y. Zhang , L. Wang , J. Zhang , J. Luo , X. Fu , Angew. Chem., Int. Ed. 2023, 62, 202302950. 10.1002/anie.202302950 36946249

[12] L. Gong , J. Energy Chem. 2018, 27, 1618.

[13] B. Zhao , J.‐W. Liu , Y.‐R. Yin , D. Wu , J.‐L. Luo , X.‐Z. Fu , J. Mater. Chem. A 2019, 7, 25878.

[14] F. Yang , J. Ye , L. Gao , J. Yu , Z. Yang , Y. Lu , C. Ma , Y. Zeng , H. Huang , Adv. Energy Mater. 2023, 13, 2301408.

[15] W. Qiao , L. Yu , J. Chang , F. Yang , L. Feng , Chin. J. Catal. 2023, 51, 113.

[16] A. Xu , S.‐F. Hung , A. Cao , Z. Wang , N. Karmodak , J. E. Huang , Y. Yan , A. Sedighian Rasouli , A. Ozden , F.‐Y. Wu , Z.‐Y. Lin , H.‐J. Tsai , T.‐J. Lee , F. Li , M. Luo , Y. Wang , X. Wang , J. Abed , Z. Wang , D.‐H. Nam , Y. C. Li , A. H. Ip , D. Sinton , C. Dong , E. H. Sargent , Nat. Catal. 2022, 5, 1081.

[17] A. Pei , R. Xie , L. Zhu , F. Wu , Z. Huang , Y. Pang , Y.‐C. Chang , G. Chai , C.‐W. Pao , Q. Gao , C. Shang , G. Li , J. Ye , H. Zhu , Z. Yang , Z. Guo , J. Am. Chem. Soc. 2025, 147, 3185. 39806308 10.1021/jacs.4c12665 PMC11803621

[18] W. Zhong , Y. Liu , D. Zhang , J. Phys. Chem. C 2012, 116, 2994. 10.1021/jp208571d 22145640

[19] J. Wang , B. Zhang , W. Guo , L. Wang , J. Chen , H. Pan , W. Sun , Adv. Mater. 2023, 35, 2211099. 10.1002/adma.202211099 36706444

[20] Y. Xu , M. Liu , M. Wang , T. Ren , K. Ren , Z. Wang , X. Li , L. Wang , H. Wang , Appl. Catal., B 2022, 300, 120753.

[21] Y. Hao , D. Yu , S. Zhu , C.‐H. Kuo , Y.‐M. Chang , L. Wang , H.‐Y. Chen , M. Shao , S. Peng , Energy Environ. Sci. 2023, 16, 1100.

[22] G. Liu , W. Zhou , Y. Ji , B. Chen , G. Fu , Q. Yun , S. Chen , Y. Lin , P.‐F. Yin , X. Cui , J. Liu , F. Meng , Q. Zhang , L. Song , L. Gu , H. Zhang , J. Am. Chem. Soc. 2021, 143, 11262. 34281338 10.1021/jacs.1c05856

[23] A. R. Poerwoprajitno , L. Gloag , J. Watt , S. Cheong , X. Tan , H. Lei , H. A. Tahini , A. Henson , B. Subhash , N. M. Bedford , B. K. Miller , P. B. O'Mara , T. M. Benedetti , D. L. Huber , W. Zhang , S. C. Smith , J. J. Gooding , W. Schuhmann , R. D. Tilley , Nat. Catal. 2022, 5, 231.

[24] H. Liao , J. Zhu , Y. Hou , Nanoscale 2014, 6, 1049. 24292647 10.1039/c3nr05590f

[25] A. A. Dubale , Y. Zheng , H. Wang , R. Hübner , Y. Li , J. Yang , J. Zhang , N. K. Sethi , L. He , Z. Zheng , W. Liu , Angew. Chem., Int. Ed. 2020, 59, 13891. 10.1002/anie.202004314 32356333

[26] M. Li , K. Duanmu , C. Wan , T. Cheng , L. Zhang , S. Dai , W. Chen , Z. Zhao , P. Li , H. Fei , Y. Zhu , R. Yu , J. Luo , K. Zang , Z. Lin , M. Ding , J. Huang , H. Sun , J. Guo , X. Pan , W. A. Goddard , P. Sautet , Y. Huang , X. Duan , Nat. Catal. 2019, 2, 495.

[27] J. Chen , Y. Ma , T. Huang , T. Jiang , S. Park , J. Xu , X. Wang , Q. Peng , S. Liu , G. Wang , W. Chen , Adv. Mater. 2024, 36, 2312369. 10.1002/adma.202312369 38581648

[28] C. Pei , S. Chen , T. Zhao , M. Li , Z. Cui , B. Sun , S. Hu , S. Lan , H. Hahn , T. Feng , Adv. Mater. 2022, 34, 2200850. 10.1002/adma.202200850 35429007

[29] A. Herzog , M. Rüscher , H. S. Jeon , J. Timoshenko , C. Rettenmaier , U. Hejral , E. M. Davis , F. T. Haase , D. Kordus , S. Kühl , W. Frandsen , A. Bergmann , B. R. Cuenya , Energy Environ. Sci. 2024, 17, 7081.

[30] H. Qiao , M. T. Saray , X. Wang , S. Xu , G. Chen , Z. Huang , C. Chen , G. Zhong , Q. Dong , M. Hong , H. Xie , R. Shahbazian‐Yassar , L. Hu , ACS Nano 2021, 15, 14928. 34423972 10.1021/acsnano.1c05113

[31] N. Nie , D. Zhang , Z. Wang , Y. Qin , X. Zhai , B. Yang , J. Lai , L. Wang , Small 2021, 17, 2102879. 10.1002/smll.202102879 34337859

[32] X. Zhang , Z. Su , L. Jiang , S. Wang , H. Gai , Z. Deng , Y. Chen , Z. Zhang , W. Zhu , Z. Zhao , X. Li , X. Zhang , J. Colloid Interface Sci. 2025, 690, 137265. 40080928 10.1016/j.jcis.2025.137265

[33] J. Theerthagiri , K. Karuppasamy , S. J. Lee , R. Shwetharani , H.‐S. Kim , S. K. K. Pasha , M. Ashokkumar , M. Y. Choi , Light: Sci. Appl. 2022, 11, 250. 35945216 10.1038/s41377-022-00904-7 PMC9363469

[34] Y. Peng , J. Cao , Y. Sha , W. Yang , L. Li , Z. Liu , Light: Sci. Appl. 2021, 10, 168. 34408125 10.1038/s41377-021-00603-9 PMC8373902

[35] X. Fan , W. Chen , L. Xie , X. Liu , Y. Ding , L. Zhang , M. Tang , Y. Liao , Q. Yang , X. Fu , S. Luo , J. Luo , Adv. Mater. 2024, 36, 2313179. 10.1002/adma.202313179 38353598

[36] D. Sun , Y. Wang , K. J. T. Livi , C. Wang , R. Luo , Z. Zhang , H. Alghamdi , C. Li , F. An , B. Gaskey , T. Mueller , A. S. Hall , ACS Nano 2019, 13, 10818. 31469544 10.1021/acsnano.9b06019

[37] V. Vivier , A. Regis , G. Sagon , C. Cachet‐Vivier , Electrochim. Acta 2001, 46, 907.

[38] X. Yuan , B. Jiang , M. Cao , C. Zhang , X. Liu , Q. Zhang , F. Lyu , L. Gu , Q. Zhang , Nano Res. 2020, 13, 265.

[39] S. Han , Y. Ma , Q. Yun , A. Wang , Q. Zhu , H. Zhang , C. He , J. Xia , X. Meng , L. Gao , W. Cao , Q. Lu , Adv. Funct. Mater. 2022, 32, 2208760.

[40] J. Zhu , L. Xia , R. Yu , R. Lu , J. Li , R. He , Y. Wu , W. Zhang , X. Hong , W. Chen , Y. Zhao , L. Zhou , L. Mai , Z. Wang , J. Am. Chem. Soc. 2022, 144, 15529. 35943197 10.1021/jacs.2c03982

[41] X. Zhang , L. Hui , D. Yan , J. Li , X. Chen , H. Wu , Y. Li , Angew. Chem., Int. Ed. 2023, 62, 202308968. 10.1002/anie.202308968 37581223

[42] S. Zhang , Z. Zeng , Q. Li , B. Huang , X. Zhang , Y. Du , C.‐H. Yan , Energy Environ. Sci. 2021, 14, 5911.

[43] X. Yang , Q. Wang , S. Qing , Z. Gao , X. Tong , N. Yang , Adv. Energy Mater. 2021, 11, 2100812.

[44] K. Guo , D. Fan , J. Bao , Y. Li , D. Xu , Adv. Funct. Mater. 2022, 32, 2208057.

[45] X. Lao , X. Liao , C. Chen , J. Wang , L. Yang , Z. Li , J. Ma , A. Fu , H. Gao , P. Guo , Angew. Chem., Int. Ed. 2023, 62, 202304510. 10.1002/anie.202304510 37278913

[46] H. Wang , C. Gao , Z. Liu , B. Li , Y. Dok Kim , J. Feng , K. Sun , Z. Peng , J. Colloid Interface Sci. 2025, 678, 1004. 39276509 10.1016/j.jcis.2024.09.004

[47] Q. Yang , S. Zhang , F. Wu , L. Zhu , G. Li , M. Chen , A. Pei , Y. Feng , J. Energy Chem. 2024, 90, 327.

[48] Z. Zhang , J. Liu , J. Wang , Q. Wang , Y. Wang , K. Wang , Z. Wang , M. Gu , Z. Tang , J. Lim , T. Zhao , F. Ciucci , Nat. Commun. 2021, 12, 5235. 34475400 10.1038/s41467-021-25562-y PMC8413426

[49] X. Wang , M. Xie , F. Lyu , Y.‐M. Yiu , Z. Wang , J. Chen , L.‐Y. Chang , Y. Xia , Q. Zhong , M. Chu , H. Yang , T. Cheng , T.‐K. Sham , Q. Zhang , Nano Lett. 2020, 20, 7751. 32959660 10.1021/acs.nanolett.0c03340

[50] X. Zhang , A. V. Kirilin , S. Rozeveld , J. H. Kang , G. Pollefeyt , A. Chojecki , B. Vanchura , M. Blum , ACS Catal. 2022, 12, 3868.

[51] X. Zhang , M. Zhang , Y. Deng , M. Xu , L. Artiglia , W. Wen , R. Gao , B. Chen , S. Yao , X. Zhang , M. Peng , J. Yan , A. Li , Z. Jiang , X. Gao , S. Cao , C. Yang , A. J. Kropf , J. Shi , J. Xie , M. Bi , J. A. Van Bokhoven , Y.‐W. Li , X. Wen , M. Flytzani‐Stephanopoulos , C. Shi , W. Zhou , D. Ma , Nature 2021, 589, 396. 33473229 10.1038/s41586-020-03130-6

[52] Z. Yu , C. Si , A. P. LaGrow , Z. Tai , W. A. Caliebe , A. Tayal , M. J. Sampaio , J. P. S. Sousa , I. Amorim , A. Araujo , L. Meng , J. L. Faria , J. Xu , B. Li , L. Liu , ACS Catal. 2022, 12, 9397.

[53] K. Xu , L. Liang , T. Li , M. Bao , Z. Yu , J. Wang , S. M. Thalluri , F. Lin , Q. Liu , Z. Cui , S. Song , L. Liu , Adv. Mater. 2024, 36, 2403792. 10.1002/adma.202403792 38742953

[54] Q. Zhou , P. Chen , J. Pan , X. Tian , Y. Yan , Int. J. Hydrogen Energy 2024, 55, 1495.

[55] X. Wu , Y. Zhang , Y. Yang , G. Fu , F. Si , J. Chen , M. Ahmad , Z. Zhang , C. Ye , J. Zhang , X.‐Z. Fu , J.‐L. Luo , Chem. Eng. J. 2023, 452, 139057.

[56] Q. Zhao , B. Zhao , X. Long , R. Feng , M. Shakouri , A. Paterson , Q. Xiao , Y. Zhang , X.‐Z. Fu , J.‐L. Luo , Nano‐Micro Lett. 2024, 16, 80. 10.1007/s40820-023-01282-4 PMC10784266 38206434

[57] Y. Yang , R. Zou , J. Gan , Y. Wei , Z. Chen , X. Li , S. Admassie , Y. Liu , X. Peng , Green Chem. 2023, 25, 4104.

