# Supplementary material for: Femtosecond‐Laser Synthesized PtBi Nanoalloys for Efficient Methanol Oxidation in Hybrid Electrolysis
Source: Adv Sci (Weinh). 2025 Aug 18;12(42):e10123. doi: 10.1002/advs.202510123 (PMC12622524; doi:10.1002/advs.202510123)
Supplement: Supplementary file 1 — Supporting Information [file ADVS-12-e10123-s001.docx]

Supporting Information

Femtosecond-Laser Synthesized PtBi Nanoalloys for Efficient Methanol Oxidation in Hybrid Electrolysis

Xianze Zhang, Zikang Su, Chen Zhang, Zhiyi Sun, Lan Jiang, Zhi Wang, Ruichen Lu, Qimiao Zhu, Shucheng Shi, Yunhong Luo, Yang Gu, Zhi Liu, Wenxing Chen*, Hui Zhang*, and Xueqiang Zhang*

X. Zhang, Z. Su, C. Zhang, L. Jiang, Z. Wang, R. Lu, Q. Zhu, X. Zhang

Laser Micro/Nano-Fabrication Laboratory, School of Mechanical Engineering, Beijing Institute of Technology, Beijing 100081, China

E-mail: xueqiangzhangme@bit.edu.cn

Z. Sun, W. Chen

Energy & Catalysis Center, School of Materials Science and Engineering, Beijing Institute of Technology, Beijing 100081, China

E-mail: [wxchen@bit.edu.cn](mailto:wxchen@bit.edu.cn)

S. Shi, Y. Luo, Y. Gu, Z. Liu

School of Physical Science and Technology, ShanghaiTech University, Shanghai 201210, China

S. Shi, Z. Liu

Center for Transformative Science, ShanghaiTech University, Shanghai 201210, China

H. Zhang

Shanghai Synchrotron Radiation Facility, Shanghai Advanced Research Institute, Chinese Academy of Sciences, Shanghai 201204, China

E-mail: zhanghui2023@sari.ac.cn;

H. Zhang

National Key Laboratory of Materials for Integrated Circuits, Shanghai Institute of Microsystem and Information Technology, Chinese Academy of Sciences, Shanghai 200050,

**Materials and Methods**

**Materials**

Bismuth nitrate pentahydrate, chloroplatinic acid (H_2_PtCl_6_·6H_2_O, 98%), Nafion (from DuPont, 5 wt%), methanol (from MREDA, CH_3_OH, 99.99%), n-hydride (MREDA), potassium hydroxide (KOH), carbon black (Cabot, XC-72R), Nafion D-521 dispersion (5 wt%, Sinero) and ethanol (EtOH) were purchased from Chengdu Kelong Chemical Reagent Co., Ltd. Commercial 20% Pt/C was purchased from Johnson Matthey. All solutions for electrochemical tests were prepared with deionized (DI) water (resistivity: 18.2 MΩ cm). All chemicals were used as received without further treatment.

**Synthesis of PtBi/C**

The catalyst synthesis process is conceptually illustrated in Figure S1. Initially, carbon black (100 mg) was dispersed in a Pt-Bi precursor mixture and sonicated for 1 hour to form a homogeneous suspension. Subsequent freeze-drying at 193 K for 6 hours, followed by grinding and immersion in hexane to suppress metal oxidation through phase immiscibility. Fs laser irradiation (800 nm, 1 kHz repetition rate) was performed using a plano-convex lens with magnetic stirring. This technique enabled non-equilibrium diffusion via ultrafast energy injection, facilitating the photoreduction of Pt^4+^/Bi^3+^ ions and forming metastable PtBi alloys. Critically, this approach circumvents the limitations of traditional thermodynamic Pt-Bi phase diagrams, which typically require prolonged high-temperature annealing for alloy synthesis. In the laboratory-scale reaction system (reaction volume 5 mL, 100 mg powder), under femtosecond lasers processing conditions (600 mW, 800 nm, and 100 fs), the yield of PtBi nanoparticles is estimated as ~133 mg/h. The total output energy of the femtosecond laser system was kept at 3.6 W. By adopting laser beam-splitting, the yield can be further increased by approximately 6 times to 798 mg/h, without affecting the quality and uniformity of the PtBi nanoparticles.

**Material characterization**

The morphology was characterized using transmission electron microscopy (TEM, JEM-2100F), high-angle annular dark-field scanning transmission electron microscopy (HAADF-STEM), and energy-dispersive X-ray spectroscopy (EDS) for elemental mapping. X-ray photoelectron spectroscopy (XPS) data were collected on a Versaprobe III system, calibrated to the C 1s peak at 284.8 eV. X-ray diffraction (XRD) patterns were recorded on a SmartLab SE diffractometer with Cu Kα radiation (λ = 1.5418 Å, 40 kV, 30 mA) from 10° to 80° 2θ. Inductively coupled plasma optical emission spectroscopy (ICP-OES) was performed on a Thermo Fisher iCAP PRO. High-resolution scanning transmission electron microscopy (HR-STEM) images were obtained using a Themis Z aberration-corrected microscope.

**Electrochemical Measurements**

Electrochemical measurements were performed using a three-electrode system on a CHI660E workstation (Chenhua, Shanghai, China). A graphite rod and Hg/HgO electrode served as the counter and reference electrodes, respectively. Catalysts were drop-cast onto 5 mm diameter glassy carbon electrodes (0.196 cm²). Potentials were calibrated to the reversible hydrogen electrode (RHE) using E_RHE_ = E_Hg/HgO_ + 0.059 pH + 0.098 V (in alkaline media). Catalyst inks were prepared by ultrasonically mixing 4 mg catalyst, 125 µL deionized water, 375 µL ethanol, and 50 µL Nafion. Then, 7.0 µL of ink was applied to the electrode and air-dried for 2 hours before measurements.

Prior to testing, catalysts were activated in Ar-saturated 1 M KOH + 1 M CH_3_OH by cycling between -0.9 and 0.3 V vs. Hg/HgO at 200 mV s^-1^ until stable CV curves were obtained. For MOR evaluation, CV curves were recorded in N₂-saturated 1 M KOH and 1 M KOH + 1 M CH_3_OH at 50 mV s^-1^. Stability was assessed via chronoamperometry at -0.15 V vs. Hg/HgO.

The two-electrode structure is assembled in a single electrolytic cell. Both the anode and cathode use 5 mm diameter glassy carbon electrodes. Different catalysts were used to form the anode and cathode. The assembled two-electrode configuration includes Pt_4_Bi/C || Pt/C and Pt/C || Pt/C. The electrolytes were 1M KOH, 1M KOH + 1M CH_3_OH, and 1M KOH + 1M CH_3_OH + natural seawater. Their polarization curves were measured at a scan rate of 50 mV s^-1^.

The Faradaic efficiency (FE) was determined using a gas collection method in a gas-tight H-cell. The evolved H₂ gas product was collected using a Burette. The FE was calculated based on the total charge passed through the electrode (Q) and the amount of H₂ produced (n) using the following equation:

$$\mathrm{FE}=\frac{2F ⨯ n}{Q}$$

where F represents the Faraday’s constant (96485 C mol^−1^).

**CO Stripping Experiment**

CO stripping was performed in 1 M KOH. The solution was first purged with high-purity N_2_ (99.999%) for 30 min, then bubbled with CO (99.9%) for 15 min to saturate the active sites with CO. Residual CO was removed by N_2_ purging for 30 min, followed by recording the CV curve at 50 mV s^-1^.

**Electrochemical Active Surface Area (ECSA) Evaluation**

ECSA was determined using underpotential deposition of copper (Cu UPD). Cu stripping was performed in N_2_-saturated 2 mM CuSO_4_ + 0.05 M H_2_SO_4_. Electrodes were first cleaned in N_2_-saturated 0.05 M H_2_SO_4_, then transferred to Ar-saturated 2.0 mM CuSO_4_ + 0.05 M H_2_SO_4_ and held at 0.31 V vs. RHE for 100 s to deposit a Cu monolayer. CV curves were then recorded from 0.31 to 1.2 V vs. RHE at 20 mV s^-1^. ECSA was calculated as ECSA = Q / (0.42 mC cm^-2^ × m_Pt_), where Q is the integrated charge from Cu stripping and m_Pt_ is the Pt mass on the electrode.

Stability of MOR-assisted seawater electrolysis was assessed via chronopotentiometry at 10 mA cm^-2^ in alkaline natural seawater. Seawater from the Bohai Sea was filtered to remove visible impurities. Mixing with 1 M KOH produced white precipitates (primarily Ca(OH)_2_ and Mg(OH)_2_), which were removed by centrifugation. The solution was then bubbled with CO₂ for 20 min to precipitate residual Ca^2+^ and Mg^2+^, and the clear supernatant was adjusted to pH 14 with KOH for use as the electrolyte.

**In-situ spectroscopic characterizations**

*In-situ* ambient pressure X-ray photoelectron spectroscopy (APXPS) was used to study the surface chemistry of PtBi catalysts under reaction conditions at the BL02B beamline, Shanghai Synchrotron Radiation Facility (SSRF). The setup isolates the gas-liquid interface via a proton exchange membrane (PEM), maintaining background pressure in the reaction chamber through permeated CH_3_OH and water vapor. In addition, 0.4 mbar of CH_3_OH was introduced into the analysis chamber to increase the coverage of CH_3_OH under reaction conditions. A 0.3 mm aperture separated the analysis chamber from the differential pumping system and hemispherical analyzer (Phoibos 150, Specs, Germany). A 100 nm silicon nitride window isolated the beam path. High-resolution spectra were recorded at 1240 eV incident energy with 20 eV resolution. C 1s, O 1s, Pt 4f, and Bi 4f spectra were collected using a Scienta Omicron hip-3 analyzer with 0.1 eV steps. High-purity CH_3_OH and H_2_O vapor were introduced via independent leak valves, and pressure was monitored with a PFEIFFER CMR 363 gauge. Spectra were acquired after 15 min of pressure stabilization.

*In-situ* Fourier transform infrared (FTIR) spectroscopy was performed using a Thermo Scientific Nicolet iS50 spectrometer equipped with a liquid-nitrogen-cooled MCT-A detector and an EverGlo IR source.

**X-ray Absorption Fine Structure (XAFS) Analysis**

X-ray absorption fine structure (XAFS) spectra were collected at the BL14W1 beamline of the Shanghai Synchrotron Radiation Facility (SSRF), operating at 3.5 GeV with a maximum current of 250 mA. Pt L_3_-edge spectra were acquired in fluorescence mode over a photon energy range of 11400-12220 eV. Multiple scans were performed to ensure reproducibility, and the averaged spectra were processed using Athena software. The energy scale was calibrated by setting the first inflection point of a Pt foil reference to 11564 eV.

EXAFS data were processed using Athena and Artemis from the IFEFFIT software suite following standard procedures. Fitting details are provided below.

The EXAFS data were processed using the ATHENA module of IFEFFIT. The spectra were generated by subtracting the post-edge background and normalizing to the edge jump. The χ(k) data were then Fourier-transformed to R-space using a Hanning window to isolate contributions from different coordination shells. Structural parameters were obtained via least-squares fitting using the ARTEMIS module ^[1]^.

The EXAFS data were fitted using the standard equation:


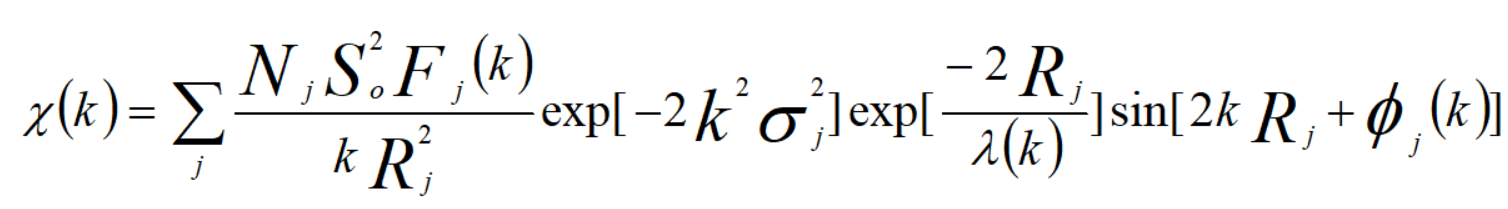


Where *S_0_^2^* is the amplitude reduction factor, *F_j_(k)* is the backscattering amplitude, *N_j_* is the coordination number, *R_j_* is the interatomic distance, *λ* is the mean free path, ϕ *_j_(k)* is the phase shift (including the phase shift for each shell and the total central atom phase shift), and *σ_j_^2^* is the Debye-Waller factor for the *j^th^* shell. *F_j_(k)*, *λ* and ϕ *_j_(k)* were calculated using FEFF8.2. Coordination numbers were fixed to nominal values, and *S_0_^2^* was was determined from reference samples and fixed in subsequent fits. While the internal atomic distances *R*, Debye-Waller factor *σ^2^*, and the edge-energy shift *ΔE_0_* were allowed to run freely.

**DFT Calculations**

Density functional theory (DFT) calculations were performed using the Vienna Ab initio Simulation Package (VASP). The generalized gradient approximation (GGA) with the Perdew-Burke-Ernzerhof (PBE) functional was employed. Ionic cores were described using projected augmented wave (PAW) potentials, with a plane wave cutoff of 450 eV. The Methfessel-Paxton smearing method (order 1, width 0.2 eV) was used for partial orbital occupancies. Geometry optimizations were conducted with a 1 × 1 × 1 Gamma-centered k-point grid, and convergence was achieved when the energy difference was less than 10^-5^ eV and atomic forces were below 0.02 eV Å^-1^. Dispersion corrections were included via the DFT + D3 method ^[2-3]^, and spin polarization was applied for magnetic systems. Gibbs free energies were calculated as G = Eelec + E_ZPE_ - TS, with E_elec_ from DFT, E_ZPE_ as the zero-point energy, and T = 298.15 K. The methanol adsorption site of PtBi-O is located on the Pt atom between the two Bi atoms.


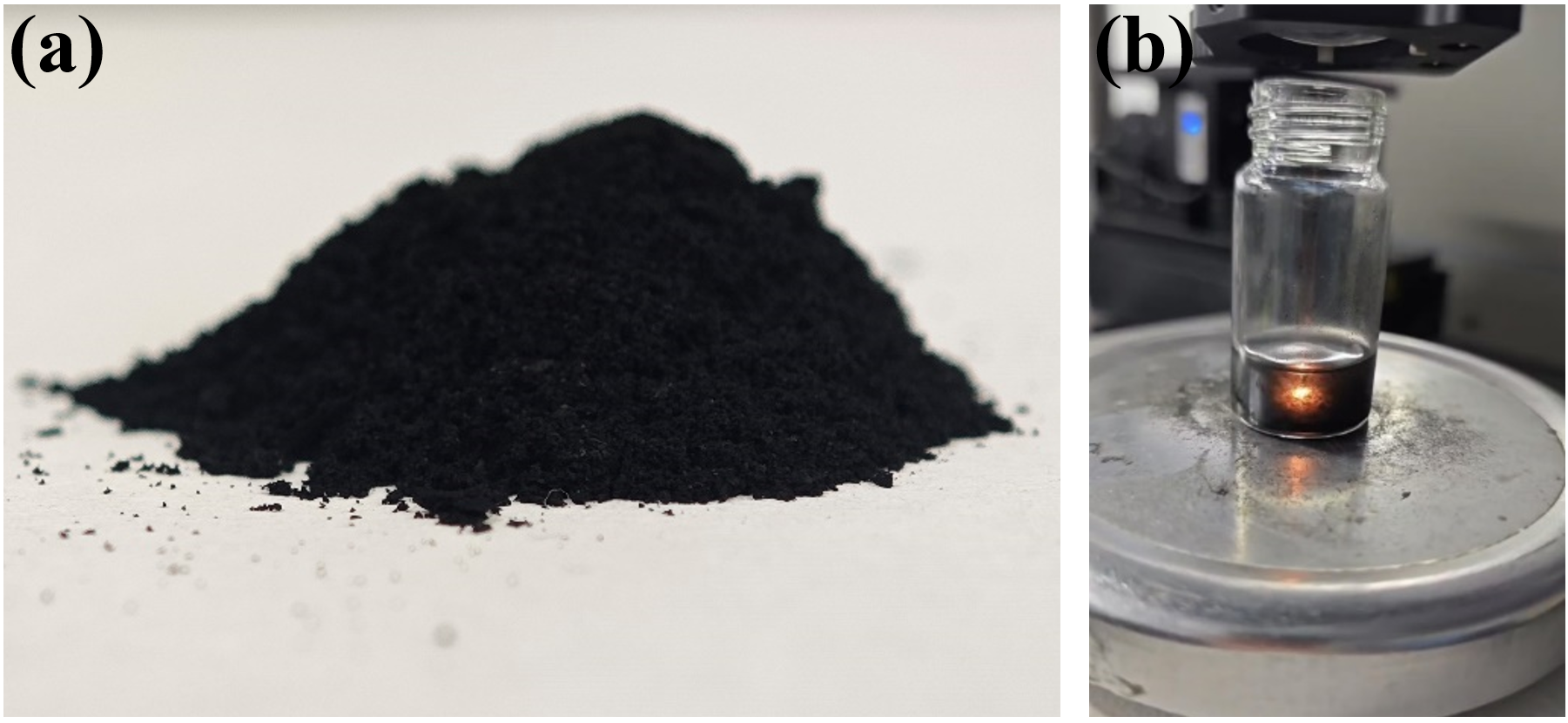


**Figure S1.** (a) Freeze-dried precursor powder. (b) Laser synthesis process in n-hexane.

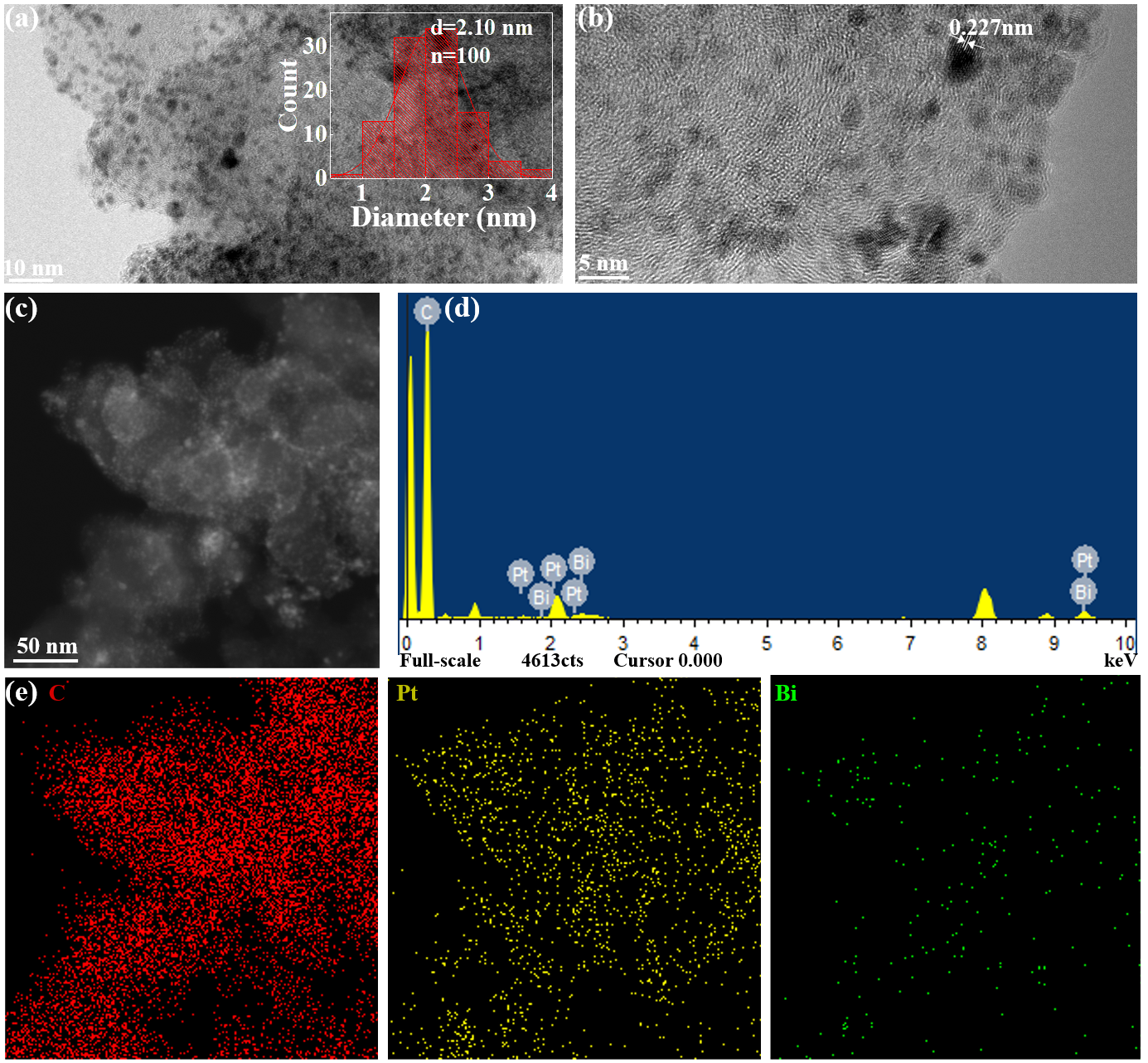


**Figure S2.** (a) TEM image of Pt_7_Bi/C. Inset: Particle size distribution histogram. (b) HRTEM image of Pt_7_Bi/C. (c) Low-magnification HAADF-STEM image of Pt_7_Bi/C. (d) EDX spectrum of Pt_7_Bi/C. (e) EDX elemental mapping of Pt_7_Bi/C.


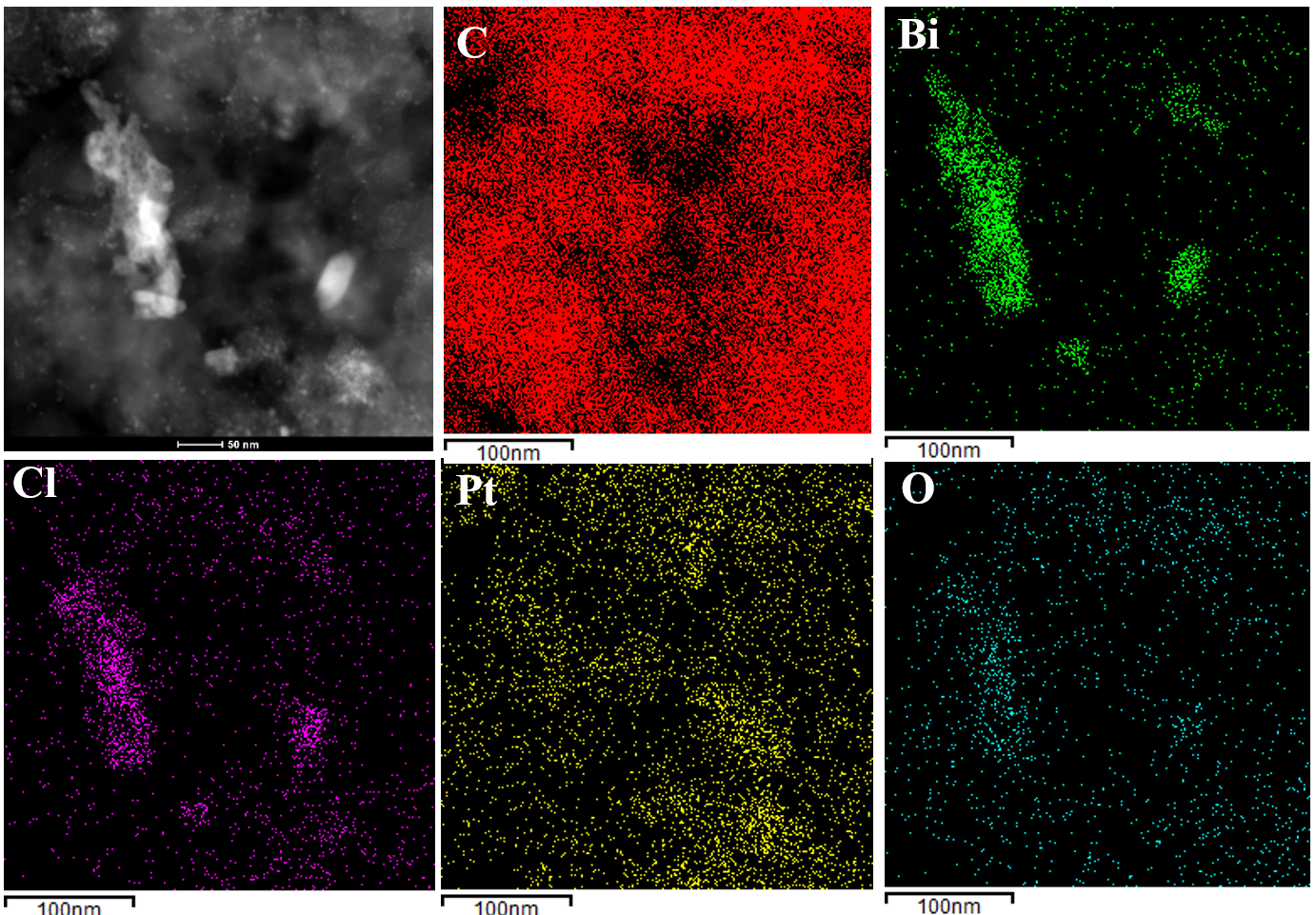


**Figure S3.** HAADF-STEM image and EDX elemental mapping of Pt_7_Bi/C.


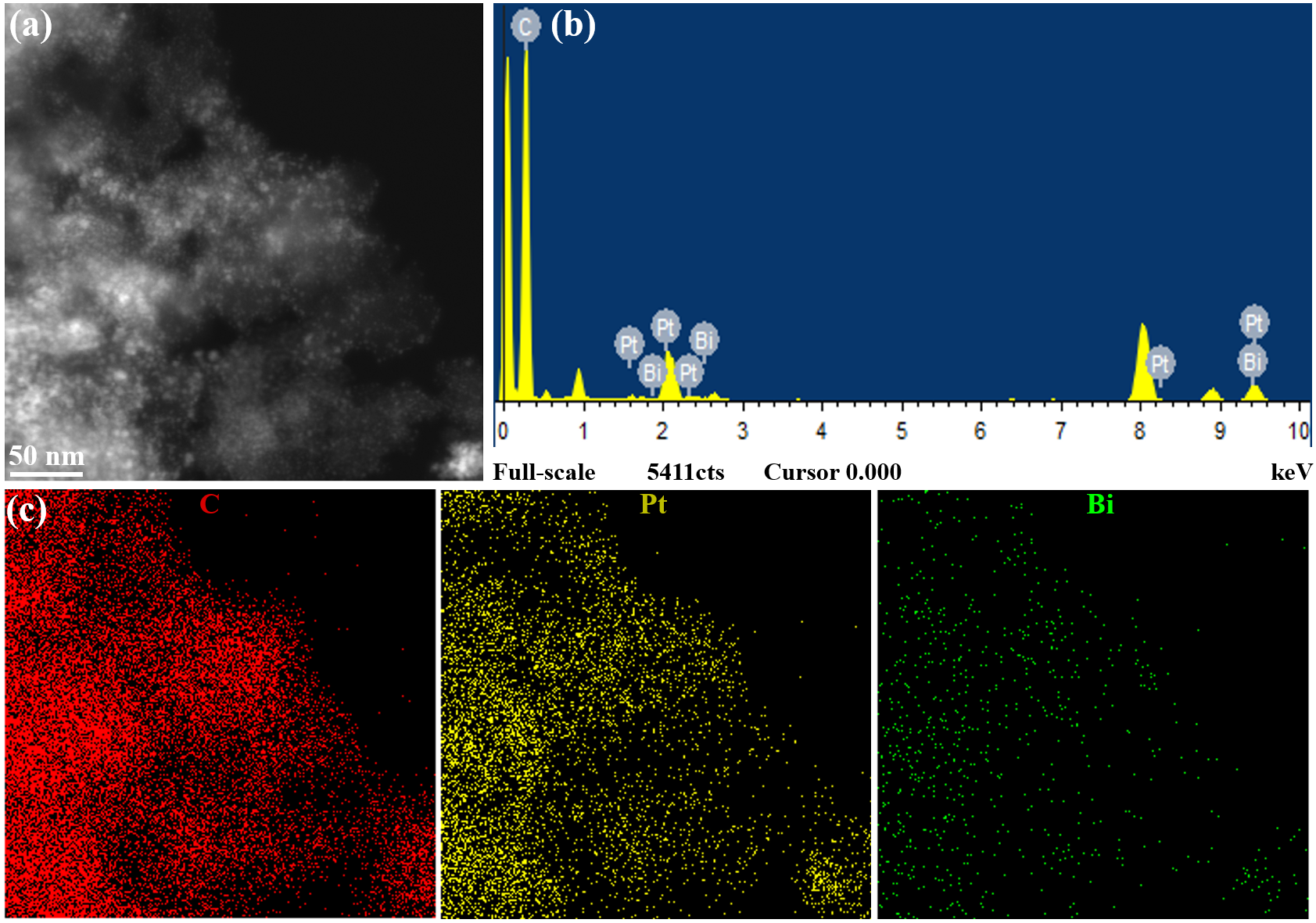


**Figure S4.** (a) HAADF-STEM image of Pt_4_Bi/C. (b) EDX spectrum of Pt_4_Bi/C. (c) EDX elemental mapping of Pt_4_Bi/C.


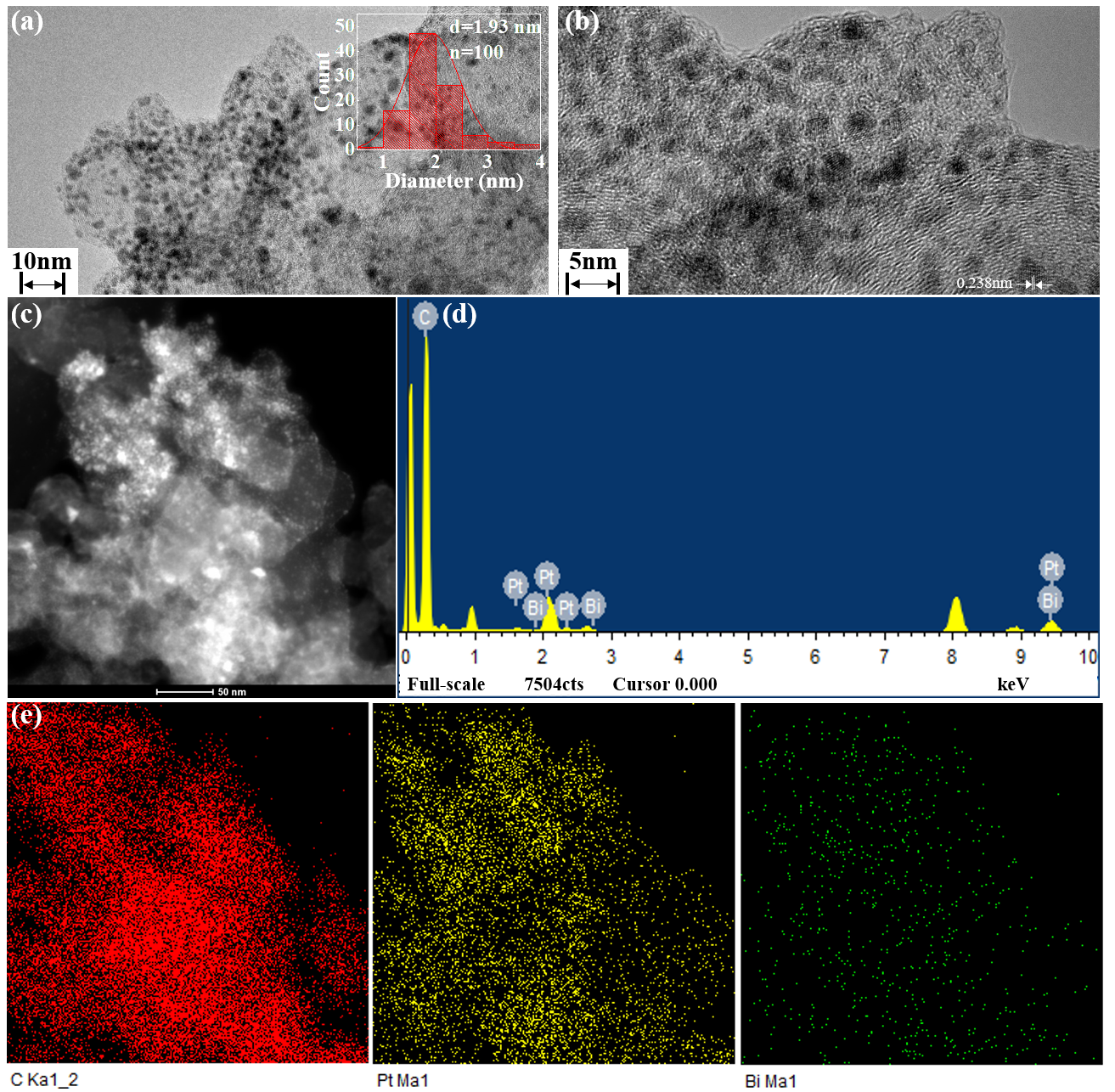


**Figure S5.** (a) TEM image of Pt_2_Bi/C. Inset: Particle size distribution histogram. (b) HRTEM image of Pt_2_Bi/C. (c) Low-magnification HAADF-STEM image of Pt_2_Bi/C. (d) EDX spectrum of Pt_2_Bi/C. (e) EDX elemental mapping of Pt_2_Bi/C.


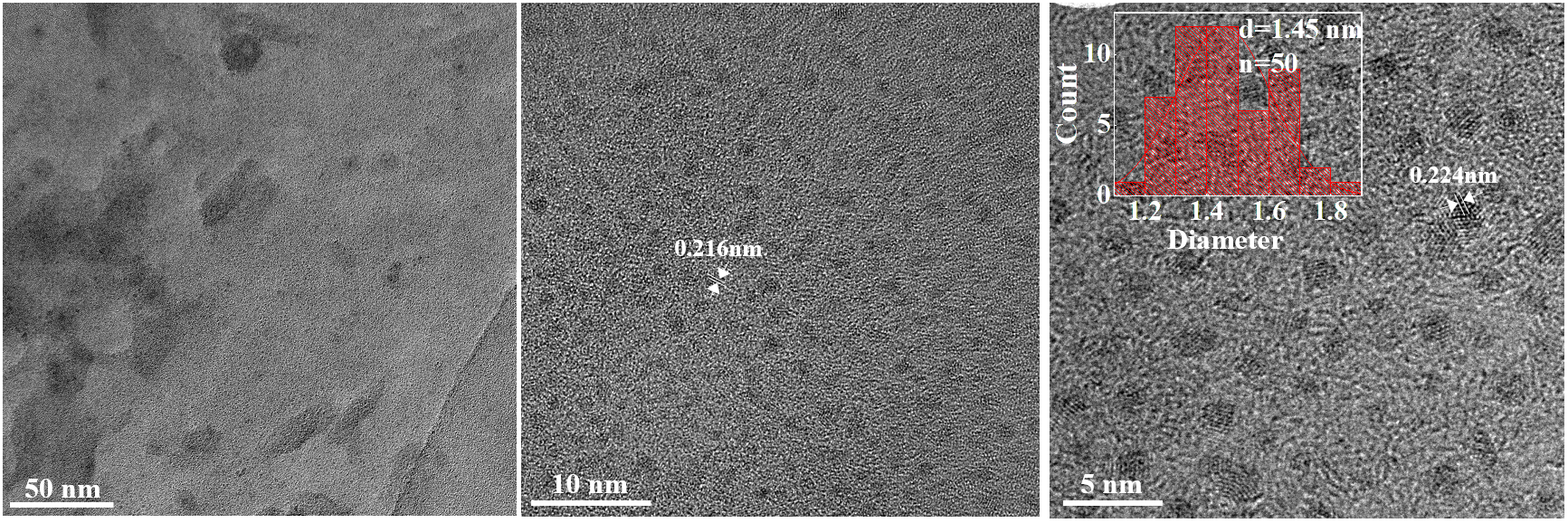


**Figure S6**. TEM images of PtRu nanoalloys.


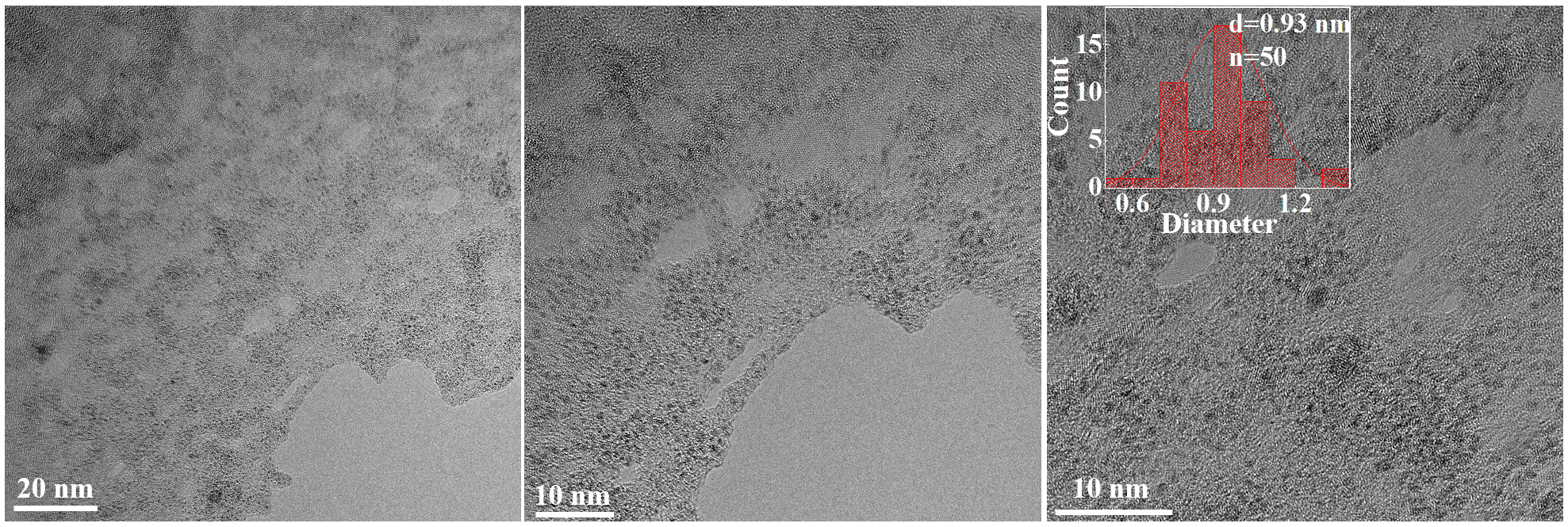


**Figure S7**. TEM images of PtRe nanoalloys.


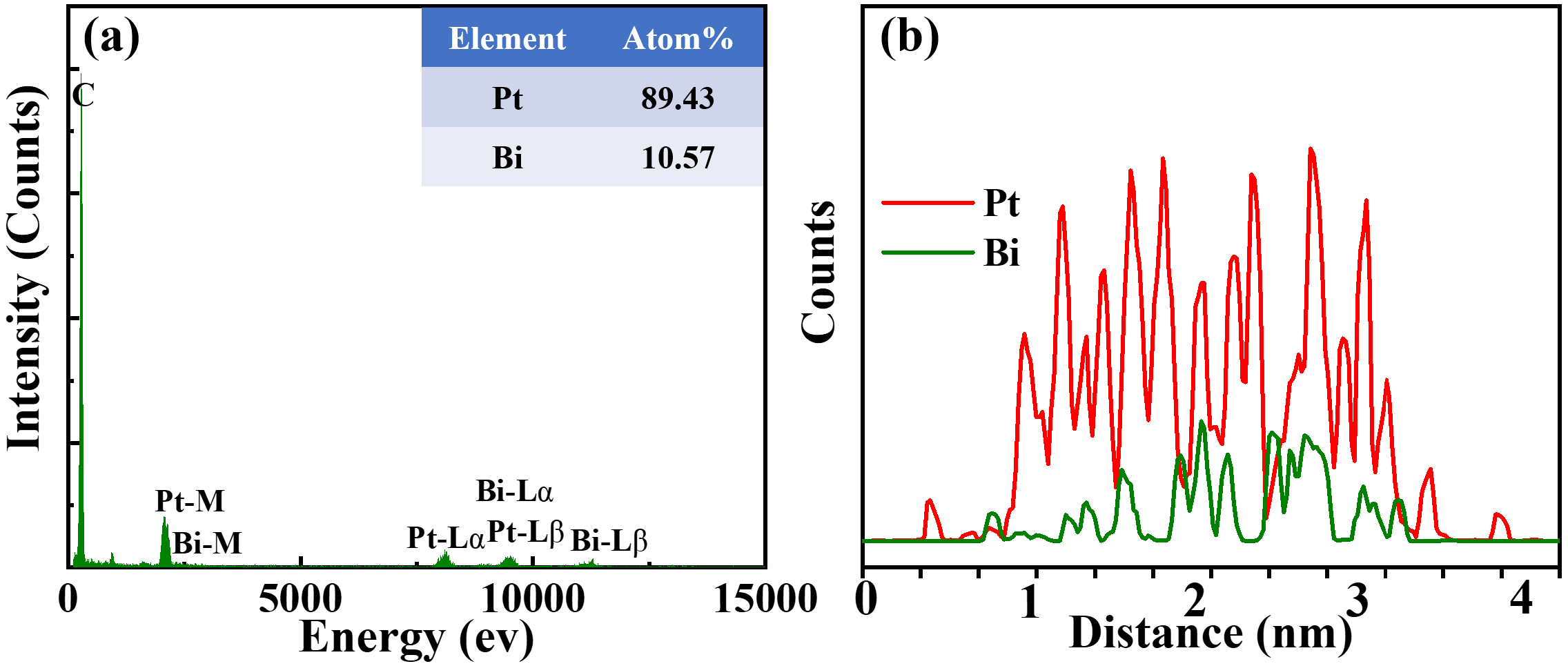


**Figure S8**. (a) Element distribution in Pt_4_Bi/C (b) Line scanning profile of Pt_4_Bi/C.


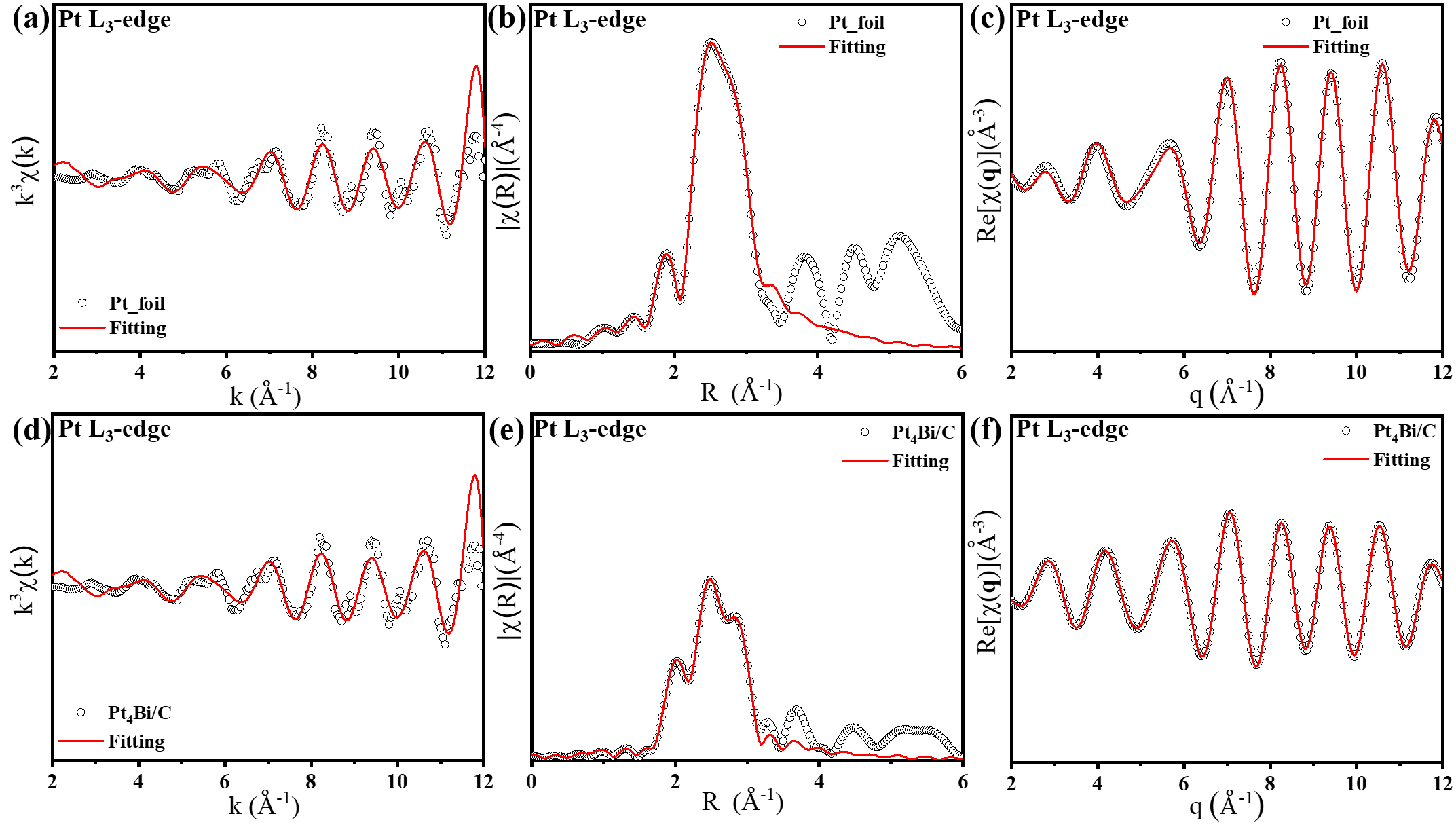


**Figure S9.** EXAFS fitting results for the Pt L_3_-edge of Pt foil and Pt_4_Bi/C.


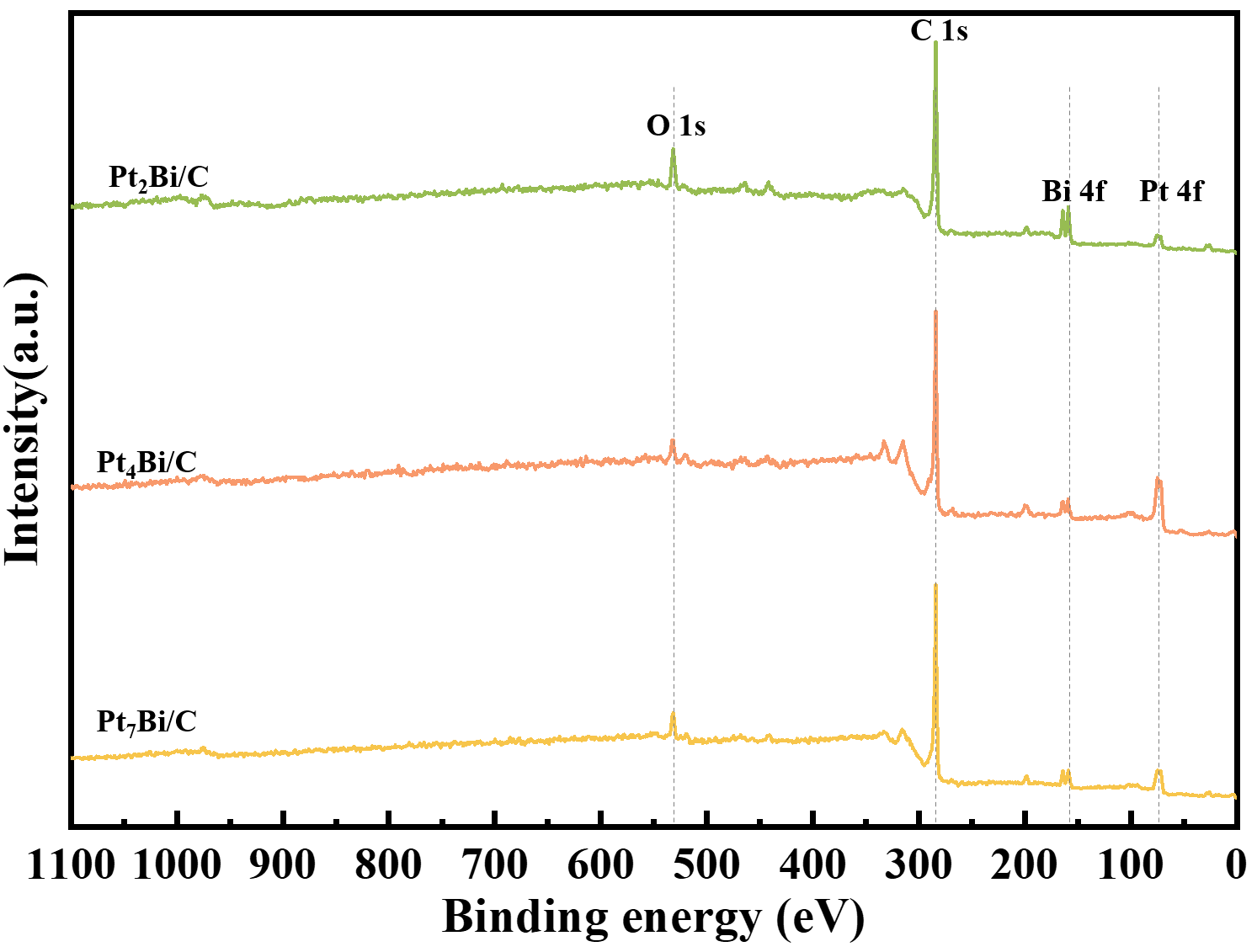


**Figure S10.** XPS survey spectra of Pt_7_Bi/C, Pt_4_Bi/C, and Pt_2_Bi/C.


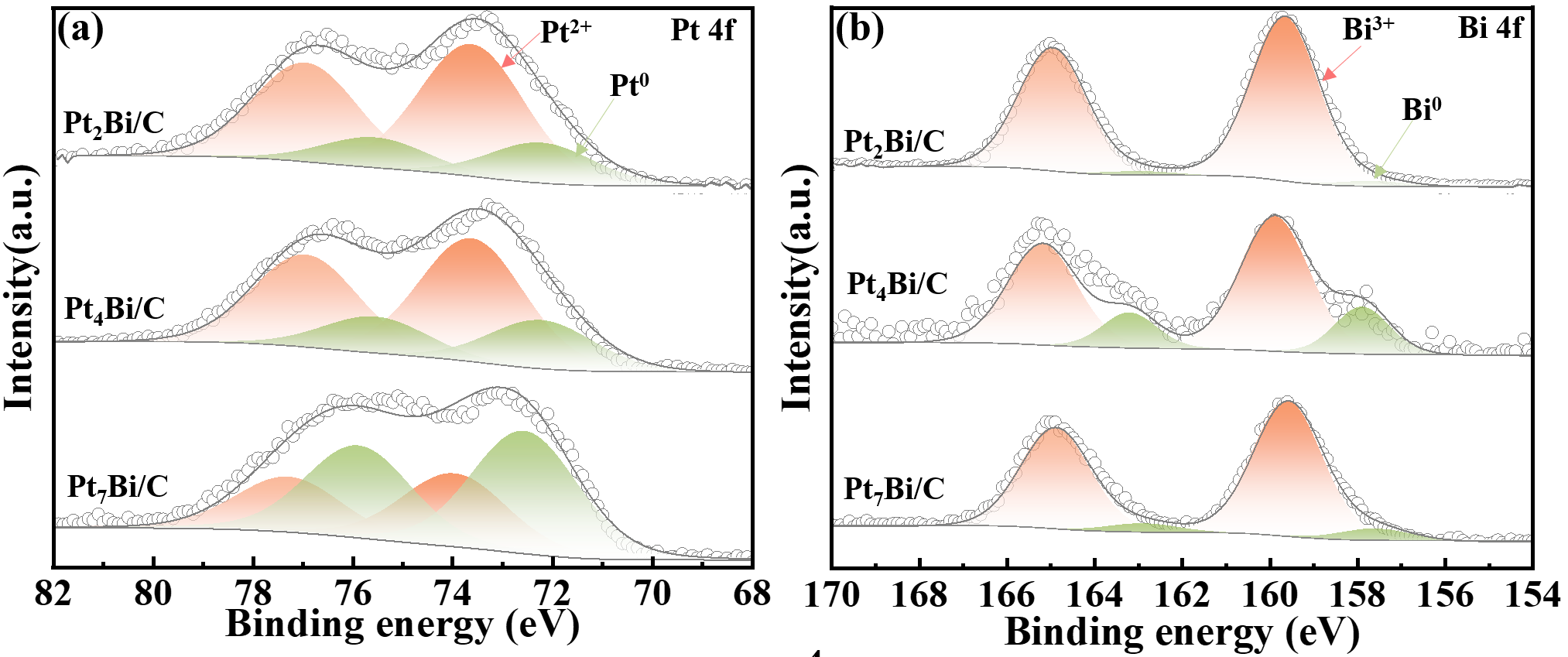


**Figure S11.** (a) Pt 4f XPS spectra of Pt_7_Bi/C, Pt_4_Bi/C, and Pt_2_Bi/C. (b) Bi 4f XPS spectra of Pt_7_Bi/C, Pt_4_Bi/C, and Pt_2_Bi/C.


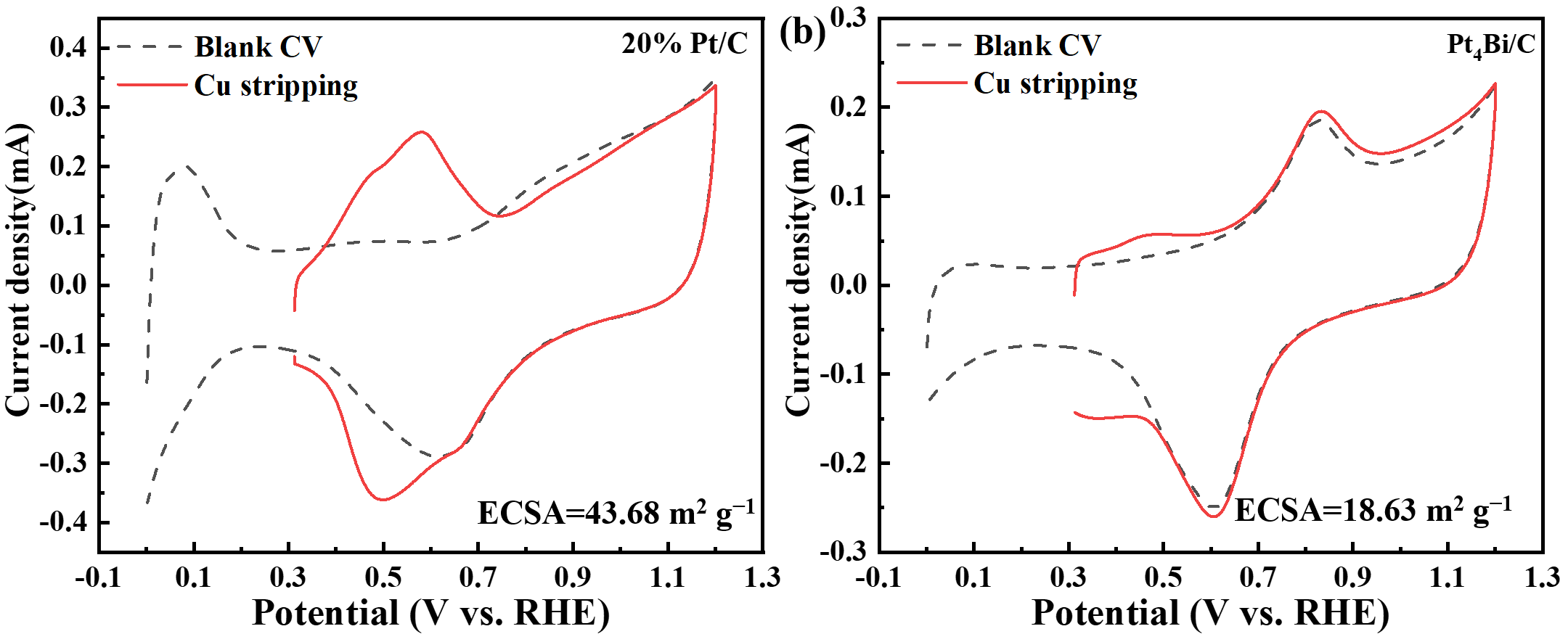
 **Figure S12.** Cyclic voltammograms and Cu stripping voltammograms for (a) 20% Pt/C and (b) Pt_4_Bi/C. CVs were recorded in 0.05 M H_2_SO_4_, and Cu stripping in 0.05 M H_2_SO_4_ + 2.0 mM CuSO_4_, both at 20 mV s^-1^.


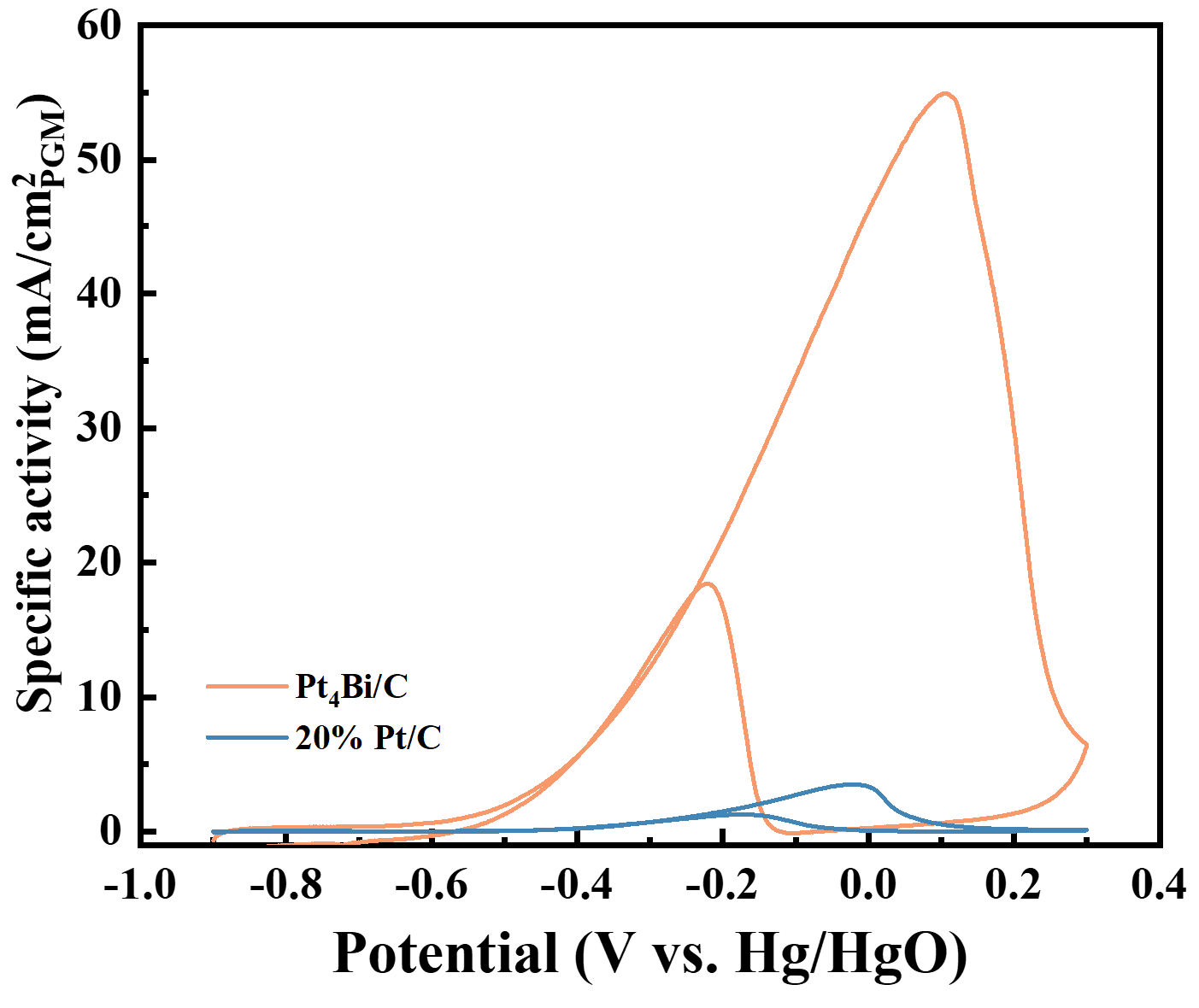


**Figure S13.** Specific activities for MOR of various catalysts in 1 M KOH + 1 M CH_3_OH at 50 mV s^-1^.


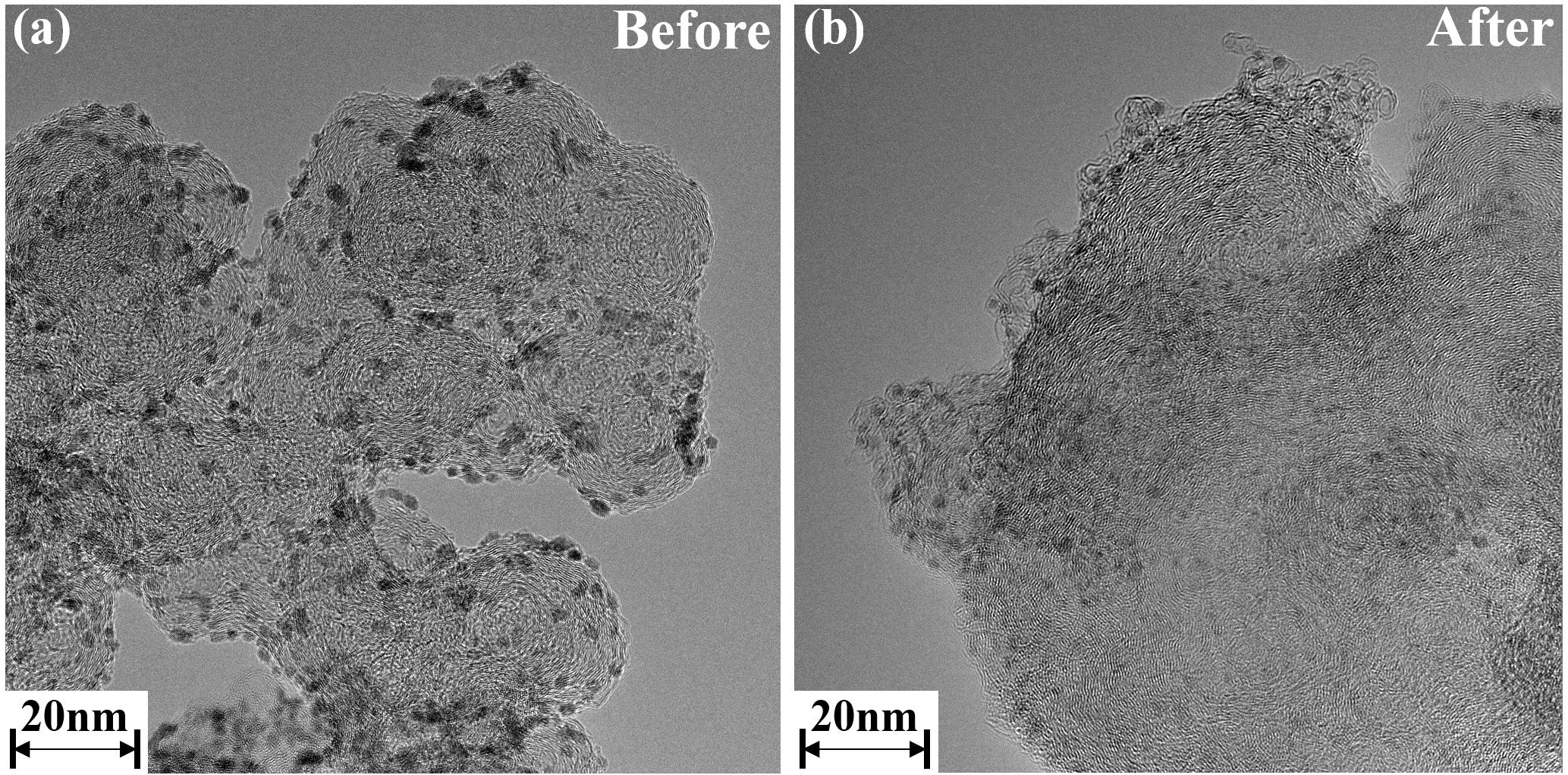


**Figure S14.** TEM images of 20% Pt/C (a) before and (b) after a 20000 s durability test.


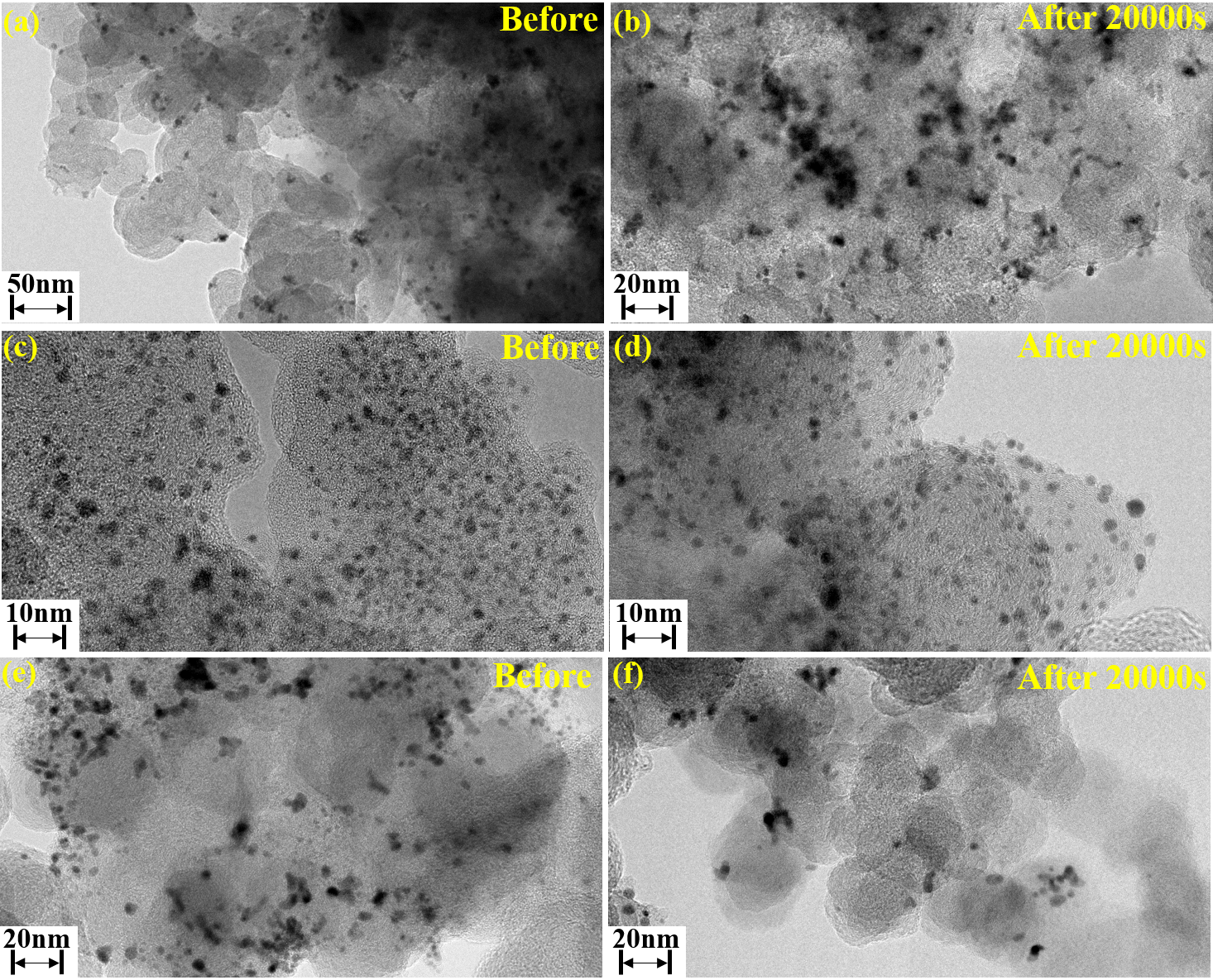


**Figure S15.** TEM images of catalysts before and after a 20000 s stability test. (a, b) Pt_7_Bi/C, (c, d) Pt_4_Bi/C, (e, f) Pt_2_Bi/C.


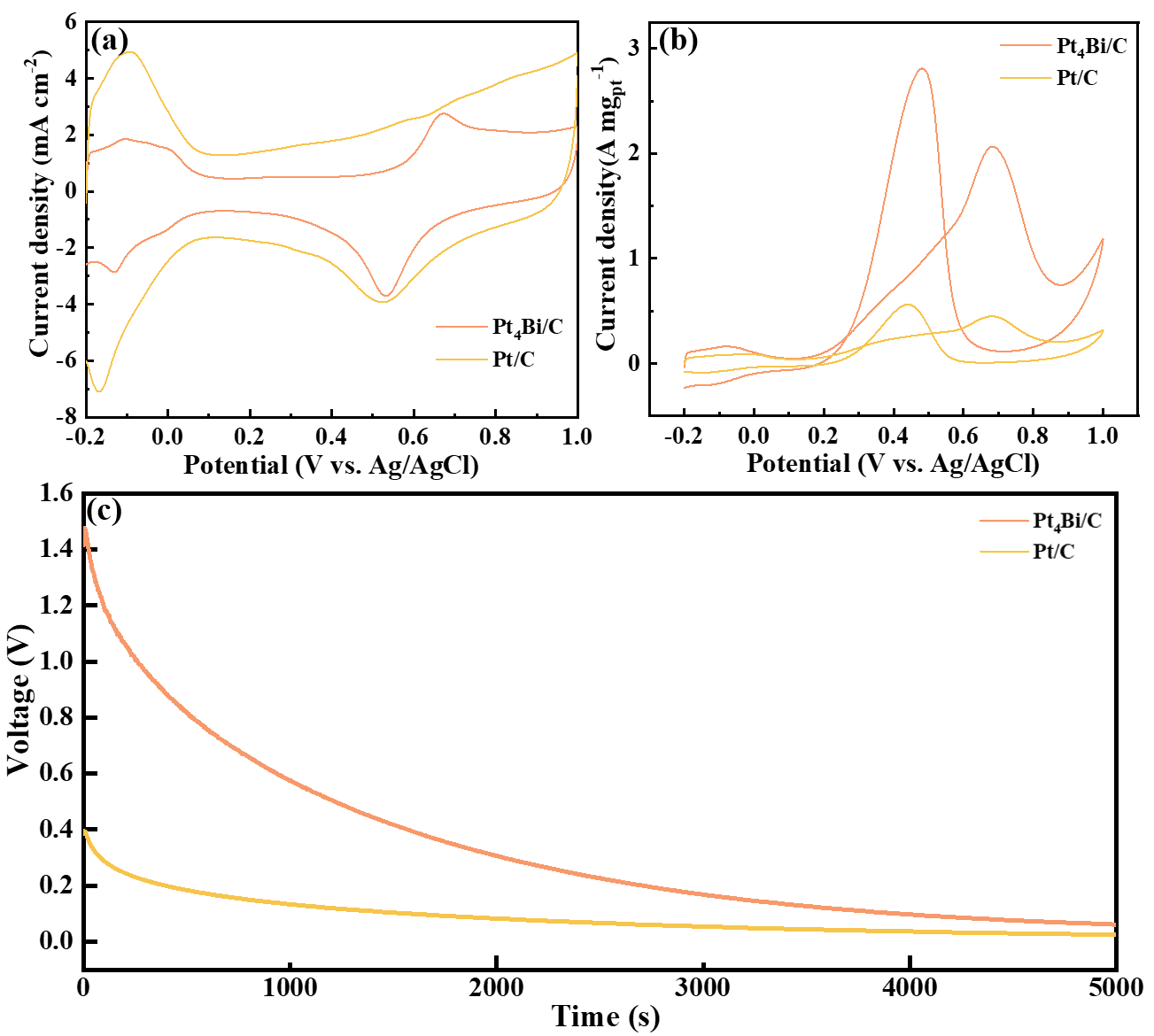


**Figure S16.** (a) CVs of Pt_4_Bi/C and commercial Pt/C catalysts in N_2_ saturated 0.5 M H_2_SO_4_ solution at a scan rate of 50 mV/s*.* (b) CVs of Pt_4_Bi/C and commercial Pt/C catalysts in N_2_ saturated 0.5 M H_2_SO_4_ solution at a scan rate of 50 mV/s. (c) Chronoamperometry curves at a constant potential of 0.6 V (vs. Ag/AgCl) in 0.5 M H_2_SO_4_ + 1 M CH_3_OH.


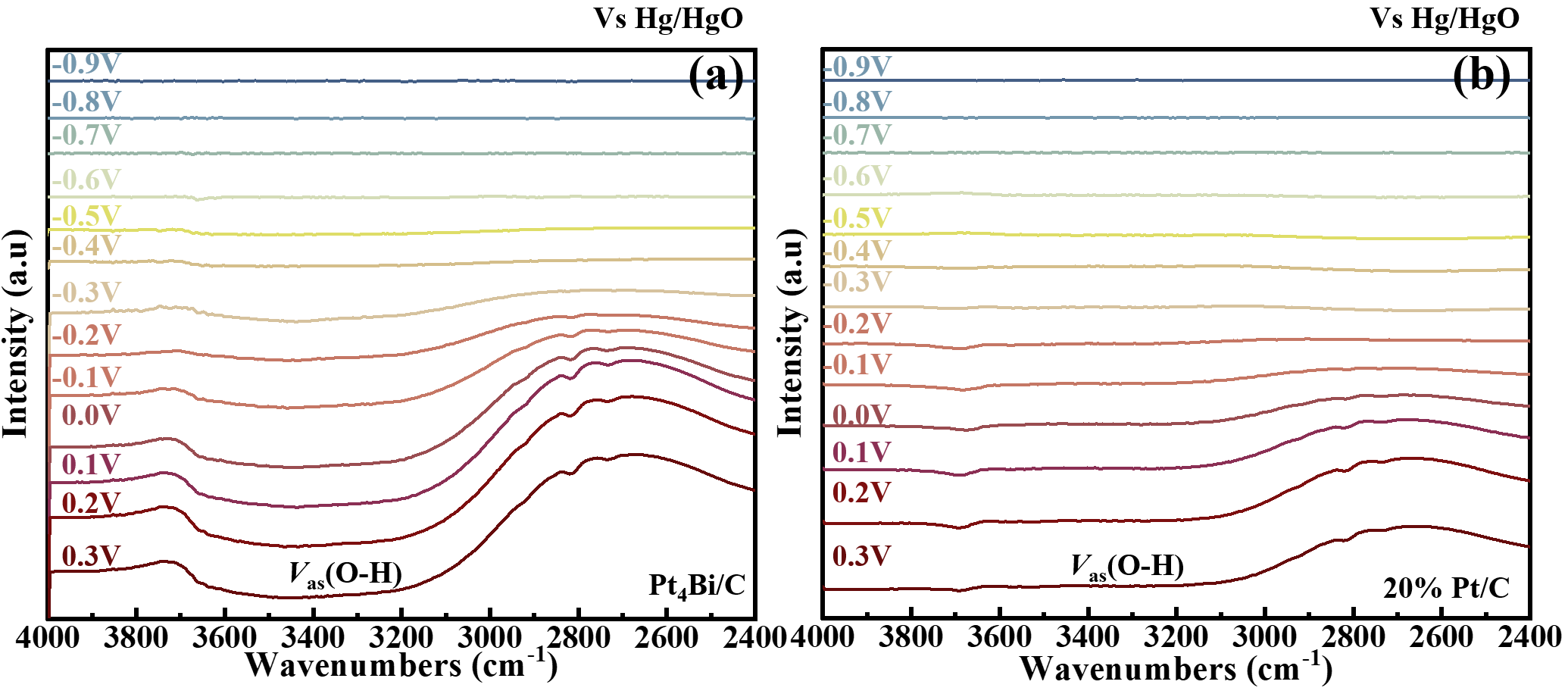


**Figure S17.** *In-situ* FTIR spectra (2400-4000 cm^-1^) of (a) Pt_4_Bi/C and (b) 20% Pt/C were recorded in a N_2_-saturated 1 M KOH + 1 M CH_3_OH.


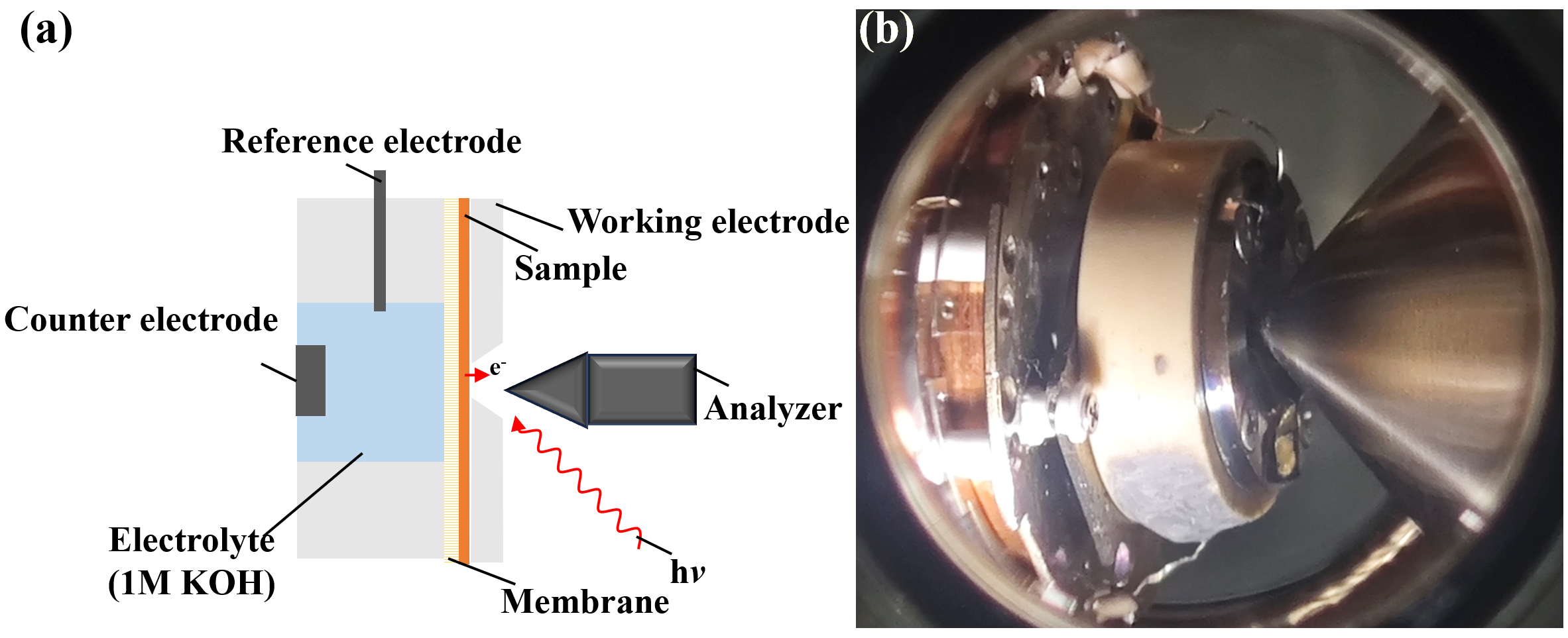


**Figure S18.** *In-situ* APXPS measurements. (a) schematic of the electrochemical cell, and (b) photograph of the electrochemical cell.


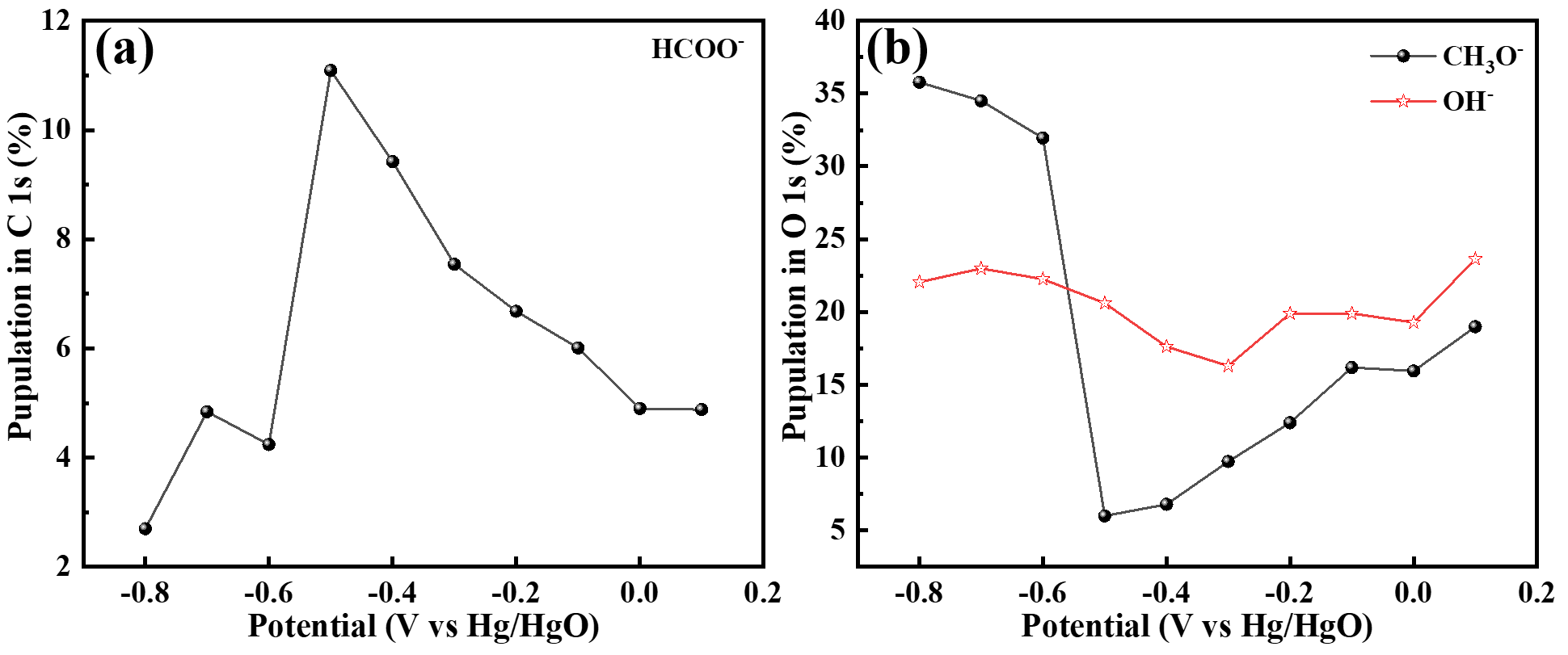


**Figure S19.** Relative contents of HCOO^-^, CH_3_O*, and OH^-^ as a function of applied potential from APXPS analysis.


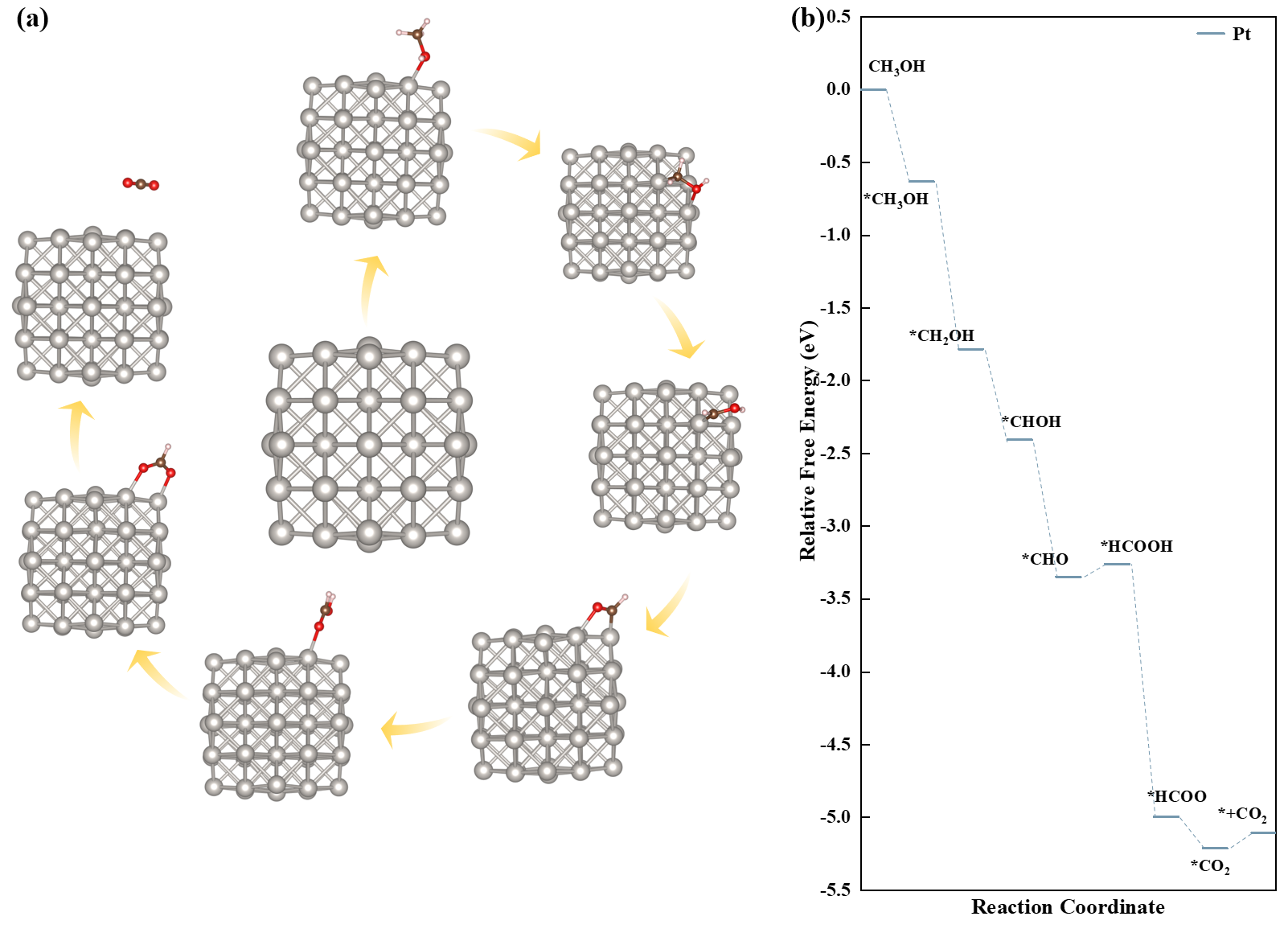


**Figure S20.** (a) Space-filling models illustrating the CO_2_ pathway in MOR on Pt clusters. (b) Free-energy profiles for MOR on Pt clusters.


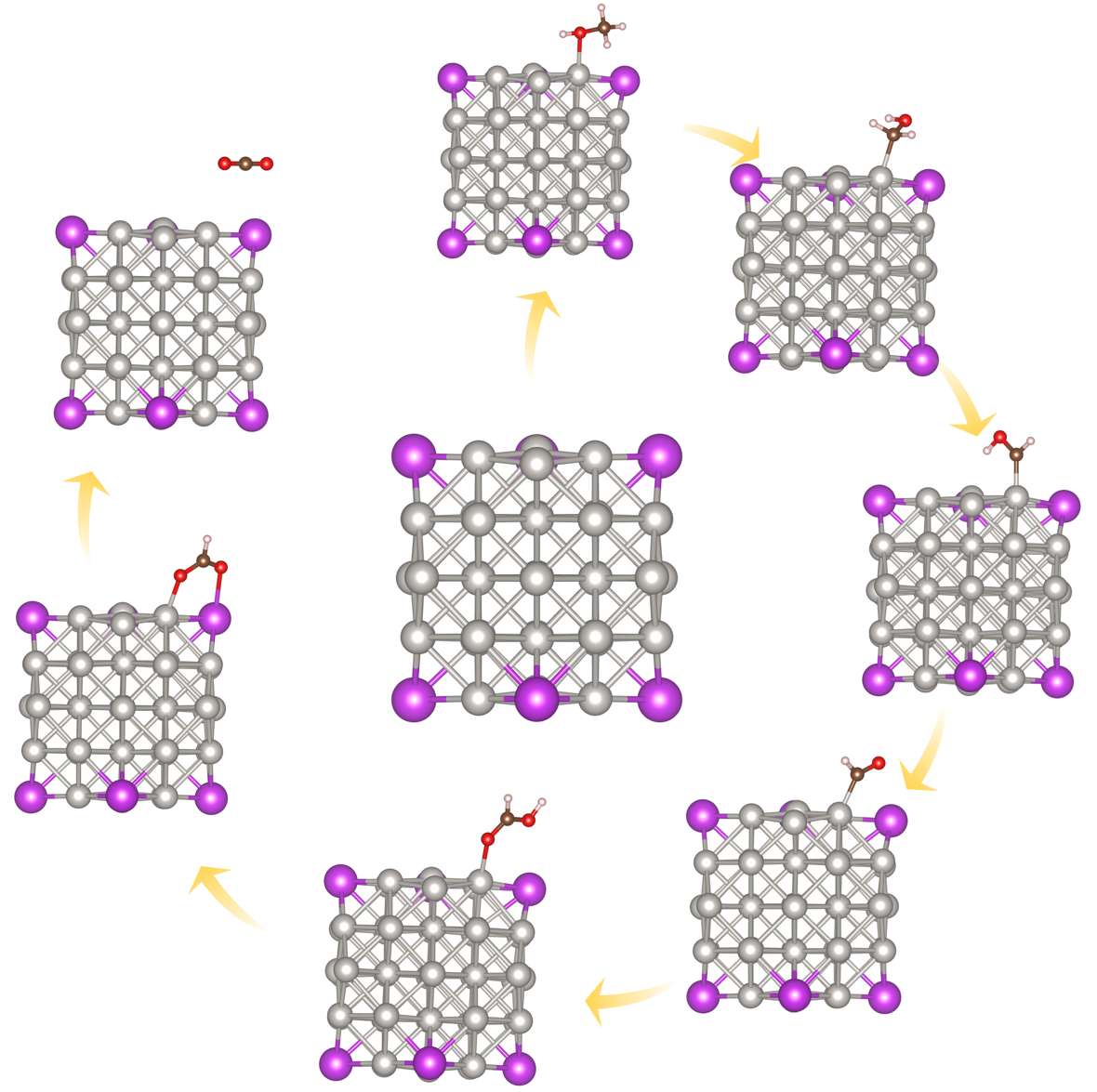


**Figure S21.** Space-filling models illustrating the CO_2_ pathway in MOR on PtBi clusters.


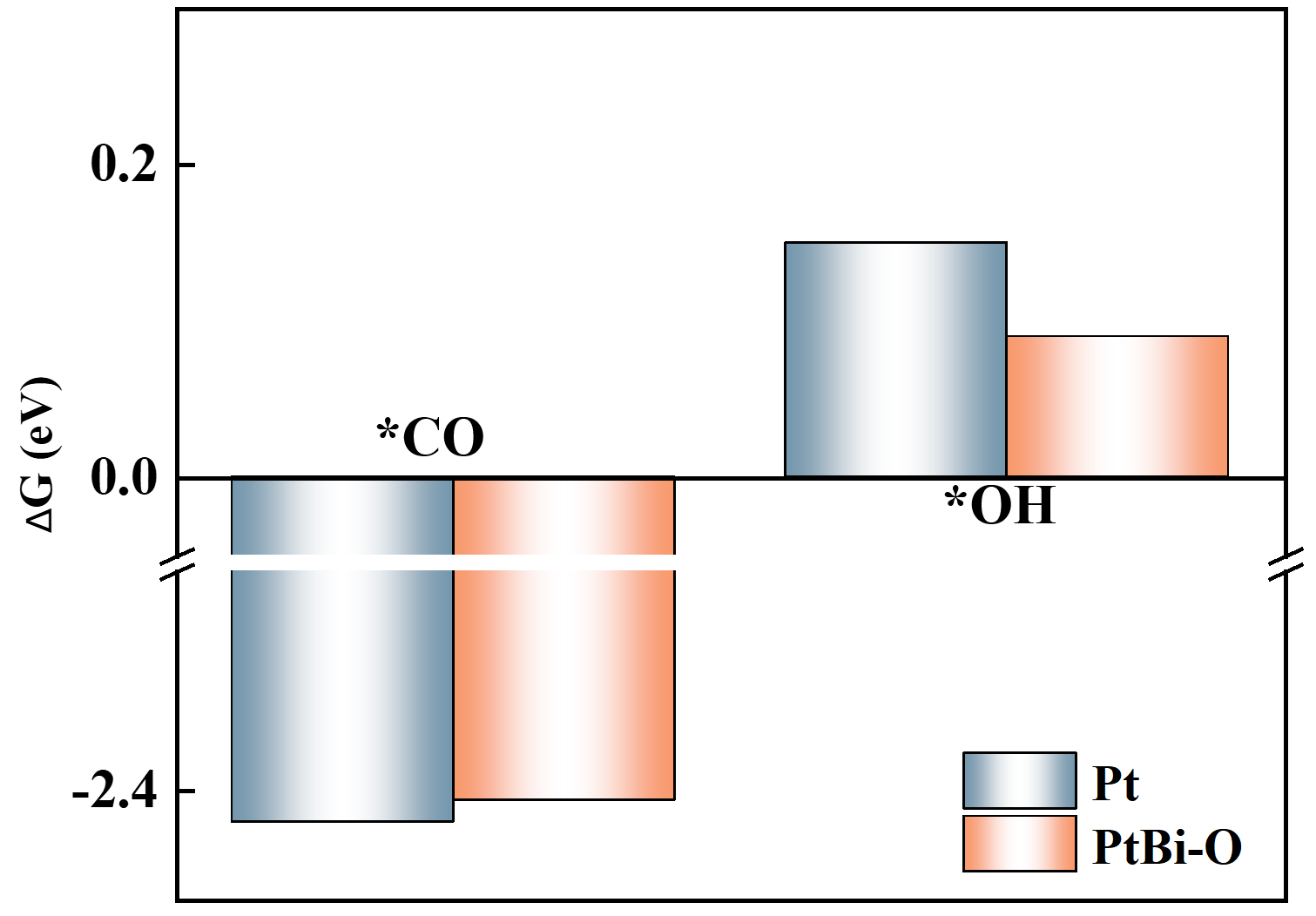


**Figure S22.** Calculated Gibbs adsorption energies of *OH and *CO on Pt and PtBi-O model catalysts.


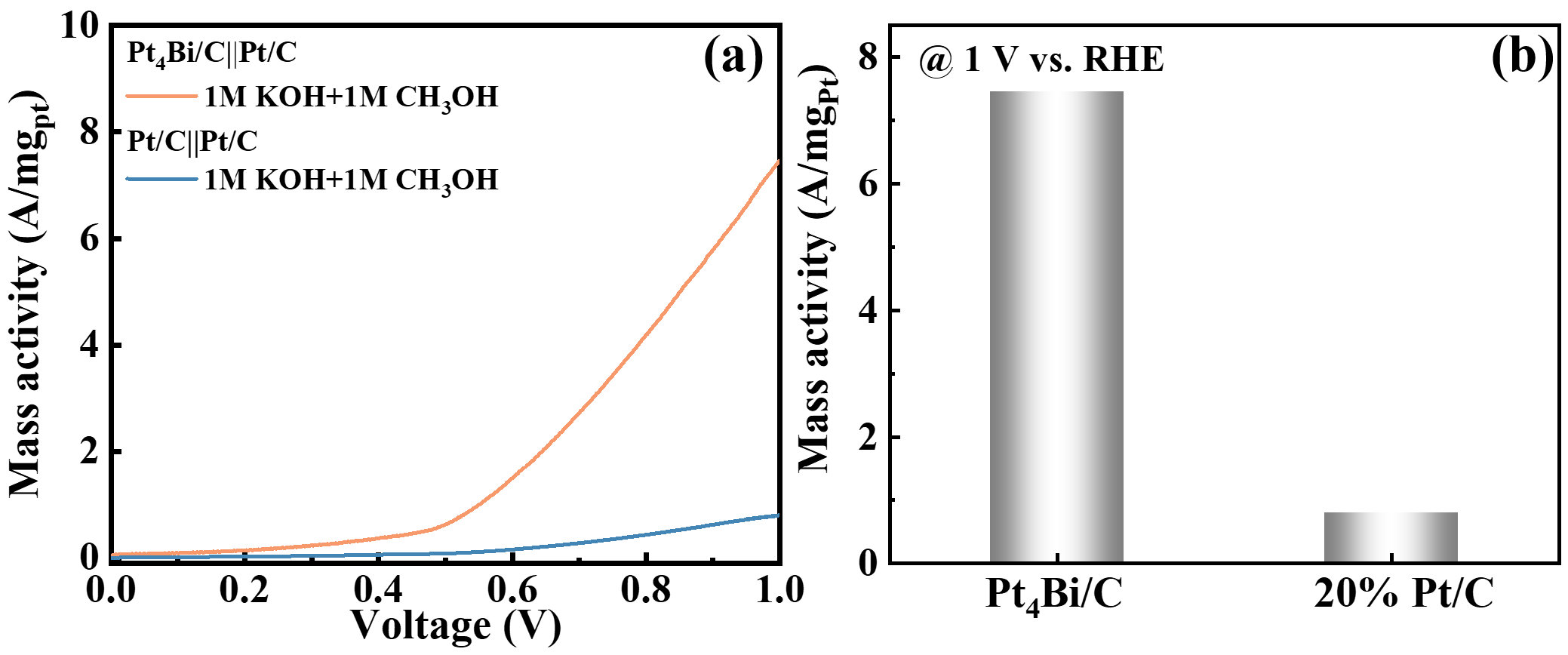


**Figure S23.** Mass activity of the two-electrode system composed of Pt_4_Bi/C and 20% Pt/C.


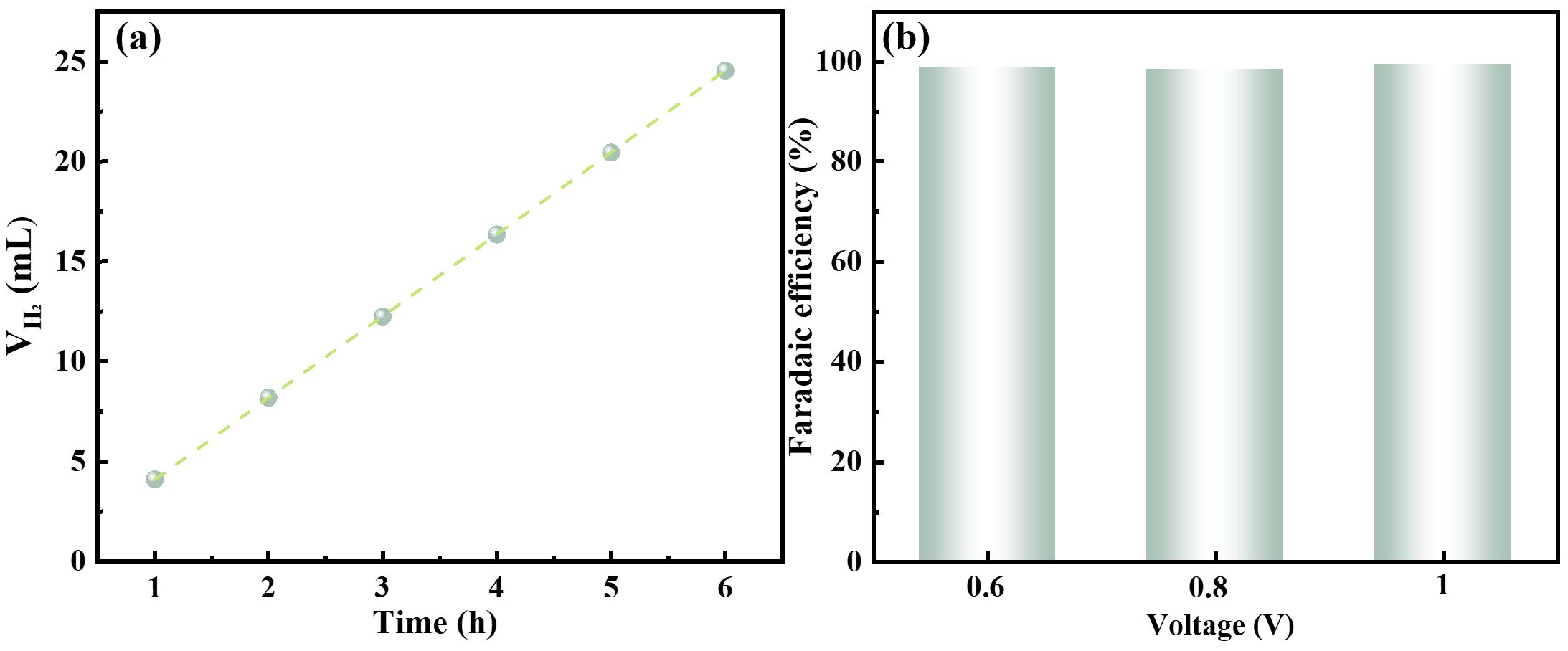


**Figure S24.** (a) The calculated theoretical values and the measured H_2_ amount, (b) Faradaic efficiency of H_2_ production in the cathodic chamber at different voltages using Pt_4_Bi/C as the anode catalysts, Pt/C as the cathode catalysts, and 1 M KOH +1 M CH_3_OH + natural seawater as the anolyte.

**Table S1.** ICP-OES analysis of of Pt_7_Bi/C, Pt_4_Bi/C, and Pt_2_Bi/C.

| Catalyst | ICP | Atomic ratio |
| --- | --- | --- |
| Pt_7_Bi/C | 9.36:1.47 | 87.2:12.8 |
| Pt_4_Bi/C | 3.95:0.94 | 81.8:18.2 |
| Pt_2_Bi/C | 4.32:2.34 | 66.4:33.6 |

**Table S2.** Fitting results of EXAFS at Pt L_3_-edge.

EXAFS fitting of Pt L-edge. (S_0_^2^ = 0.85)

| **Sample** | **Path** | **CN** | **R(Å)** | **σ^2^(10^-3^Å^2^)** | **ΔE_0_(eV)** | **R factor** |
| --- | --- | --- | --- | --- | --- | --- |
| Pt foil | Pt-Pt | 12* | 2.79 | 5.5 | 6.7 | 0.002 |
| Pt_4_Bi/C | Pt-Pt | 3.3 | 2.78 | 5.2 | 4.7 | 0.011 |
|  | Pt-Bi | 3.6 | 2.66 | 4.3 | 5.0 |  |

S_0_^2^ is the amplitude reduction factor; CN is the coordination number; R is interatomic distance (the bond length between Pt central atoms and surrounding coordination atoms); σ^2^ is Debye-Waller factor (a measure of thermal and static disorder in absorber-scatterer distances); ΔE_0_ is edge-energy shift (the difference between the zero kinetic energy value of the sample and that of the theoretical model). R factor is used to value the goodness of the fitting.

* This value was fixed during EXAFS fitting, based on the known structure.

Error bounds that characterize the structural parameters obtained by EXAFS spectroscopy were estimated as N ± 20%; R ± 1%; σ^2^ ± 20%; ΔE_0_ ± 20%.

**Table S3.** Comparison of mass activities for MOR in alkaline electrolytes among advanced electrocatalysts.

| Catalysts | Electrolyte | Scan rate  (mv s^-1^) | Mass activity (A mg^-1^) | Specific activity (A cm^-2^) | References |  |
| --- | --- | --- | --- | --- | --- | --- |
| Pt_4_Bi/C | 1.0M KOH+1.0M CH_3_OH | 50 | 17.7 | 54.9 | **This work** | |
| Pt_1.8_Pd_0.2_CuGa/C i-NPs | 1.0M KOH+1.0M CH_3_OH | 50 | 11.13 |  | Adv. Mater. 2024, 36, 2403792 | |
| 20% Pt/C | 1.0M KOH+1.0M CH_3_OH | 50 | 1.529 | 15.6 | **This work** | |
| PtBi NRs | 1.0M KOH+1.0M CH_3_OH | 50 | 6.42 | 11.93 | Adv. Funct. Mater. 2022, 32, 2208760 | |
| PdAgSn/PtBi | 1.0M KOH+1.0M CH_3_OH | 50 | 2.87 | 4.7 | Angew. Chem. Int. Ed. 2023, 62, e202304510 | |
| Ptc/Ti_3_C_2_T_x_ | 1.0M KOH+1.0M CH_3_OH | 10 | 7.32 |  | J. Am. Chem. Soc.  2022, 144, 15529–15538 | |
| A/IMC PtPbBi  NSs | 1.0M KOH+1.0M CH_3_OH | 50 | 11.9 | 14.8 | Angew.Chem. Int.Ed. 2024, 63,e202405173 | |
| Pt_1_/RuO_2_ single atom | 0.1M KOH+1M CH_3_OH | 20 | 6.77 |  | Nat.Commun.2021,12  5235 | |
| SANi-Pt NWs | 1.0M KOH+1.0M CH_3_OH | 20 | 7.93 |  | Nat. Catal. 2019,2,495 | |
| PtNiGaSnMoRe  senary NWs | 1.0M KOH+1.0M CH_3_OH | 50 | 6.2 | 12.3 | Adv. Energy Mater.  2023, 13, 2301408. | |
| Au@PdPt | 1.0M KOH+1.0M CH_3_OH | 50 | 4.83 |  | Adv. Energy Mater.  2021, 11, 2100812 | |
| Pd-UNs/Cl-GDY | 1.0M KOH+1.0M CH_3_OH | 50 | 3.6 |  | Angew. Chem. Int. Ed.  2023, 62, e202308968. | |
| Pt_5_Ce | 1.0M KOH+1.0M CH_3_OH | 50 | 9.13 | 32.74 | Energy Environ. Sci.  2021,14, 5911-5918. | |
| PtP NDs | 1.0M KOH+1.0M CH_3_OH | 50 | 4.2 | 173.3 | Adv. Func. Mater.  2022, 32, 2208057. | |
| Pt_1_Ru_3_@MCHS | 1.0M KOH+1.0M CH_3_OH | 50 | 5.86 |  | Journal of Colloid And Interface Science 678 (2025) 1004–1011 | |
| Pt_1_Fe_2_/C | 1.0M KOH+1.0M CH_3_OH | 50 | 5.4 |  | Journal of Energy Chemistry 90 (2024) 327–336 | |

**Table S4** Catalytic performance of Pt-based electrocatalysts for methanol electro-oxidation in acidic medium.

| **Electrocatalysts** | **Electrolyte** | **Scan rate**  **(mV/s)** | **Mass activity**  **(A/mg_Pt_)** | | **Ref.** | |
| --- | --- | --- | --- | --- | --- | --- |
| Pt_4_Bi/C | 0.5M H_2_SO_4_ +  1.0 M CH_3_OH | 50 | 2.06 | **This work** | |  |
| Pt/N-CNT-600 | 0.5M H_2_SO_4_ +  1.0 M CH_3_OH | 50 | 2.39 | Small 2023, 2303065 | |  |
| Pt Cu_2_/SN  MWCNT | 0.5 M H_2_SO_4_  + 0.5 M  CH_3_OH | 50 | 1.58 | Chinese.  J. Catal, 2021, 42, 1205-1215. | |  |
| PtRu/CNTs-PIL | 0.5 M H_2_SO_4_  + 0.5 M  CH_3_OH | 50 | 0.242 | Angew. Chem., Int. Ed. 2009  48, 4751-4754. | |  |
| PtPd hollow nanoparticles | 0.5 M H_2_SO_4_  + 1.0 M  CH_3_OH | 50 | 0.58 | J. Am. Chem. Soc. 2013, 135, 16762-16765. | |  |
| PtRu/rGO-2 | 0.5 M H_2_SO_4_  + 1.0 M  CH_3_OH | 50 | 0.739 | J. Colloid. Interf. Sci. 2019,  557, 729-736. | |  |
| PtZn MWNT-E | 0.5 M H_2_SO_4_  + 1.0 M  CH_3_OH | 50 | 0.612 | J. Am. Chem. Soc. 2017, 139, 4762-4768. | |  |
|  |  |  |  |  | |  |

**Table S5** Comparison of current density and cell voltage for PtBi || Pt/C and other reported catalysts.

| **Catalyst** | **Current density**  **(mA cm^-2^)** | | **Cell voltage (V)** | **Electrolyte** | **Ref.** |
| --- | --- | --- | --- | --- | --- |
| Pt_4_Bi/C \|\| 20% Pt/C | 10 | 0.545/0.572 | | 1M KOH +1M CH_3_OH/natural seawater | **This work** |
| 20% Pt/C \|\| 20% Pt/C | 10 | 0.635/0.653 | | 1M KOH +1M CH_3_OH/natural seawater | **This work** |
| Pt/NiO | 10 | 1.239 | | 1M KOH +1M CH_3_OH | Chem.Eng.J.2021,411,128292. |
| Pt NWs | 10 | 0.61 | | 1M KOH +0.5M CH_3_OH | Energy Mater. 2025, 5, 500068. |
| Pt/Cr_2_O_3_-CrN | 10 | 0.624 | | 0.5 M H_2_SO_4_+1M CH_3_OH | International Journal of Hydrogen Energy 55 (2024) 1495–1504 |
| Pt-Ni_3_S_2_ | 10 | 1.35 | | 1M KOH +1M CH_3_OH | Nano-Micro Lett. (2024) 16:80 |
| PtNi-Sev | 10 | 0.64 | | 1M KOH+0.5M CH_3_OH | SmartMat 3(1)(2022)130-141 |
| Pt/MoSe_2_ | 10 | 0.75 | | 0.5 M H_2_SO_4_+1M CH_3_OH | Chinese Journal of Catalysis 51 (2023) 113–123 |
| PtPd/rGO-2 | 10 | 0.83 | | 1M KOH+1M CH_3_OH | Nanoscale,2023,15, 16904–16913 |
| Pt-NP/NiO-NS | 10 | 1.39 | | 0.5 M H_2_SO_4_+1M CH_3_OH | Chem.Eng.J.411 (2021)128292 |
| Pt/MoP-NC | 10 | 0.7 | | 1M KOH+2M CH_3_OH | J. Mater. Chem. A 10 (2022) 2021–2026 |
| Pt/MoP-NC | 10 | 0.67 | | 0.5 M H_2_SO_4_+1M CH_3_OH | Acta Phys. -Chim. Sin. 2023, 39 (9), 2301005 |

**Table S6** Comparison of stability test duration and current density between this work and reported electrocatalysts.

| **Electrocatalysts** | **Current density**  **(mA cm^-2^)** | **Time (h)** | | **Ref.** | |
| --- | --- | --- | --- | --- | --- |
| Pt_4_Bi/C \|\| Pt/C | 10 | 54 | **This work** | |  |
| Pt/Cr_2_O_3_-CrN | 10 | 10 | International Journal of Hydrogen Energy 55 (2024) 1495–1504 | |  |
| Pt/MoSe_2_ \|\| Pt/MoSe_2_ | ~10 | 18 | Chinese Journal of Catalysis 51 (2023) 113–123 | |  |
| Vp-Ni_2_P-Pt/CC | ~17 | 6 | J. Mater. Chem. A 10 (2022) 2021–2026 | |  |
| Pt/C-KI | 5 | 80 | Applied Catalysis B: Environmental 320 (2023) 121992 | |  |
| Pt-CoSe/NC-800 | ~10 | 12 | Chemical Engineering Journal 452 (2023) 139057 | |  |
| Pt/MoP-NC | 10 | 10 | Acta Phys. -Chim. Sin. 2023, 39 (9), 2301005 | |  |
| PtPd-NF | 5 | 40 | ACS Sustainable Chemistry Engineer 6 (2018) 12367−12374 | |  |
| NiFe-LDH/NiFe  HAB/CF | 20 | 28 | Small, 2023, 19,  2208027. | |  |
| Cr-doped Ni(OH)2 | 35 | 15 | Nat. Commun. 2018, 9, 4531. | |  |
| Ni_0.33_Co_0.67_(OH)_2_/NF | 30 | 20 | ACS Appl. Energy Mater. 2023, 6, 1763-1770. | |  |
| CoxP@NiCo  LDH/NF | 45 | 20 | Adv. Funct.  Mater. 2023, 33, 2306786 | |  |
| Cu_2_O-Cu@Ni_2_P/NF | 40 | 2 | Adv. Funct.  Mater. 2023, 33, 2306786 | |  |

**References**

[1] S. Liu, X.F. Lu, J. Xiao, X. Wang, X.W. Lou, Angew Chem Int Ed. **2019**, *58*, 13828–13833.

[2] S. Grimme, S. Ehrlich, L. Goerigk, J. Comput. Chem. **2011**, *32*, 1456-1465.

[3] S. Grimme, J. Antony, S. Ehrlich, H. Krieg, J. Chem. Phys. **2010**, *132*, 154104.
